# ISAR-Mamba: A Dual-Stream Gated Mamba with Hierarchical Spatial Summaries for ISAR Image Captioning

**DOI:** 10.3390/s26185916

**Published:** 2026-09-18

**Authors:** Yonghua He, Aoxiang Pan, Yonggang Li, Jiahao Wang, Wei Qu, Weigang Zhu, Wenhang Ji, Guodian Tang, Junyi Lv

**Affiliations:** Space Engineering University, Beijing 101400, China; heyonghua1980@hgd.edu.cn (Y.H.); liyonggang@hgd.edu.cn (Y.L.); wjh_cwj@hgd.edu.cn (J.W.); quwei@hgd.edu.cn (W.Q.); zwg@hgd.edu.cn (W.Z.); jijiji0908@hgd.edu.cn (W.J.); 15216596951@163.com (G.T.); 17267108363@163.com (J.L.)

**Keywords:** Inverse Synthetic Aperture Radar (ISAR), space target, image captioning

## Abstract

**Highlights:**

**What are the main findings?**

- ISARCap 1.0 was constructed as the first ISAR image captioning dataset for space targets. It covers 38 classes of space targets, and each image is accompanied by five natural language captions.
- Benefiting from the targeted design, ISAR-Mamba outperforms existing image captioning methods on multiple metrics.

**What are the implications of the main findings?**

- ISARCap 1.0 provides an evaluation benchmark, and its construction pipeline also serves as a reference for subsequent related research.
- ISAR-Mamba suggests the feasibility of state space model (SSM) architectures for ISAR image captioning, providing an efficient linear-complexity alternative for this field.

**Abstract:**

Inverse Synthetic Aperture Radar (ISAR) imagery plays a crucial role in space situational awareness, yet its interpretation remains largely confined to tasks such as classification and segmentation, lacking the ability to generate detailed natural language captions. To address this limitation, this paper proposes ISARCap 1.0, the first dataset specifically designed for ISAR image captioning, covering 38 classes of space targets and comprising 102,965 simulated images and 514,825 image–text pairs. On this basis, this paper proposes ISAR-Mamba, a dual-stream gated state space model for ISAR image captioning. The model introduces a Dual-Stream Asymmetric Encoder (DSAE), which performs complementary patch partitioning and scanning along the range and azimuth dimensions, respectively, to accommodate the physical dimensional differences of ISAR images. Meanwhile, a Sparsity-Aware Gating Mechanism (SAGM) is introduced, which jointly suppresses the interference of empty patches on state updates by leveraging scattering energy priors and a learnable scoring module. Furthermore, a Hierarchical Spatial Summarization (HSS) strategy is proposed to extract structured summary tokens from different depths and spatial regions of the encoder, thereby enhancing visual information perception during text sequence generation. Experimental results on the ISARCap 1.0 dataset indicate that ISAR-Mamba outperforms existing image captioning methods on multiple metrics, suggesting the effectiveness of the proposed method and the usability of the dataset.

## 1. Introduction

Inverse Synthetic Aperture Radar (ISAR) [1], as an active imaging system, possesses all-day, all-weather operational capabilities and is an important method for acquiring high-resolution images of space targets. It has been widely applied in fields such as spacecraft on-orbit servicing and national defense security [2,3,4]. Traditional ISAR image interpretation has mainly focused on tasks such as target classification [5,6], recognition [7,8], and semantic segmentation [9,10]. However, as application scenarios become increasingly complex, merely outputting class labels or segmentation masks can no longer satisfy the demands of intelligent information acquisition. An important open issue is how computers can describe space target ISAR images in natural language in a way similar to human experts. Such descriptions should include target types, structural components, and their spatial relationships.

Image captioning [11,12,13], as a cross-modal task that connects computer vision and natural language processing, provides a new paradigm for high-level semantic understanding of ISAR images. This task aims to extract high-level semantic information from images and automatically generate semantically rich, structurally coherent natural language captions for a given image, which not only improves the efficiency of human–computer interaction but also provides key support for applications such as image retrieval and visual question answering. In recent years, with the rapid development of deep learning techniques, image captioning has achieved remarkable progress in the domain of natural images. Mainstream image captioning methods follow an encoder–decoder architecture, which offers the advantages of simple structure, flexible sentence length, and natural caption generation. Early methods employed convolutional neural networks (CNNs) [14,15] or vision Transformers (ViTs) [16,17] as visual encoders to obtain feature representations of images, and then used sequence models such as recurrent neural networks (RNNs) or Transformers as decoders to convert image features into natural language. However, CNNs lack the ability to model global information, particularly in extracting and representing global relationships. Compared with the local feature extraction capability of CNNs, ViTs can capture global features in images through self-attention mechanisms, but their computational complexity is proportional to the square of the image sequence length, which requires greater computational resources and storage space.

Recent studies have shown that state space models (SSMs) [18,19], particularly the Mamba model [20], possess outstanding long-sequence modeling capabilities, linear computational complexity, and efficient parallel computation, and are widely regarded as an alternative to Transformers. By introducing a data-dependent selective scan mechanism, Mamba achieves effective selection of key information and context modeling in long sequences while maintaining linear complexity, and a series of Mamba variants have achieved performance comparable to or even surpassing that of Transformers in visual tasks and text generation tasks. However, the selective scan mechanism in the original Mamba model was primarily designed for processing one-dimensional causal sequences, and it suffers from significant drawbacks when processing two-dimensional image data with non-causal structures. Directly flattening a two-dimensional image into a one-dimensional sequence destroys its inherent spatial structure, leading to the loss of correlation information between adjacent regions. To alleviate this problem, some works have focused on studying two-dimensional scan mechanisms for natural images [21,22,23,24], which predefine multiple scan directions through multiple SSM branches to fully capture the dependencies between image regions. Nevertheless, since the aforementioned methods are primarily designed for natural images, they still face numerous challenges when applied to ISAR images.

ISAR images are fundamentally different from natural images. Their imaging mechanism is complex, and the interpretation of visual information is highly challenging [25]. Moreover, these images reflect the electromagnetic scattering distribution of the target rather than its reflectance characteristics. Consequently, existing Mamba-based vision models cannot be directly transferred to the ISAR image captioning task. Specifically, this mismatch primarily stems from the following reasons. First, ISAR images are inherently highly sparse [26,27,28]; the effective information of the target is concentrated in only a few strong scattering points, while the vast majority of regions contain low-amplitude background noise or zero pixel values. If equal importance weights are assigned to all image regions, a large number of redundant tokens containing almost no effective information will be introduced into sequence propagation, thereby degrading the quality of visual encoding. Second, the physical dimensions of ISAR images are anisotropic, with the range and azimuth dimensions corresponding to different resolutions, scattering mechanisms, and target representations [29]. However, existing multi-directional scanning strategies fail to perform differentiated modeling for this physical characteristic.

In addition to the above model-level limitations, what is more critical is that there is currently no publicly available ISAR image captioning dataset. In the field of natural images, the emergence of large-scale manually annotated datasets such as MS COCO [30] and Flickr30K [31] has greatly promoted the development of natural image captioning techniques. However, in the ISAR domain, due to the sensitivity of imaging data, the high threshold of professional interpretation knowledge, and annotation costs, datasets with natural language captions are still lacking to date. This has also led to an absence of published research in the field of ISAR image captioning.

To address the above issues, this paper constructs ISARCap 1.0, the first natural language captioning dataset for space target ISAR images, comprising 102,965 images and 514,825 image–text pairs. The dataset contains ISAR images of 38 classes of space targets, with each image accompanied by five text captions with varied wording, covering key semantic information such as target type, functional role, operating orbit, main components, and their spatial relationships. The construction process of the dataset includes hierarchical template-based caption generation, prompt standardization, and manual verification, which ensures semantic accuracy, linguistic consistency, and varied wording, thereby providing a reliable training and evaluation foundation for the ISAR image captioning task.

Building upon this, this paper proposes ISAR-Mamba, a dual-stream gated SSM for ISAR image captioning. First, a Dual-Stream Asymmetric Encoder (DSAE) is designed. In light of the physical differences between the range and azimuth dimensions, it adopts distinct patch partitioning strategies and scanning orders for the two streams: the range stream employs patches that are large along the range dimension and small along the azimuth dimension, and scans along the range direction to capture the macroscopic contour of the target; the azimuth stream employs patches that are small along the range dimension and large along the azimuth dimension, and scans along the azimuth direction to perceive scattering details. This design enables the SSM sequences to extract visual features simultaneously along two complementary physical dimensions. Second, a Sparsity-Aware Gating Mechanism (SAGM) is introduced. This mechanism first extracts scattering point energy from image patches as a physical prior, and then combines it with a learnable lightweight scoring module to jointly generate importance scores. Through a differentiable gating operation, it modulates the input of the SSM, dynamically suppressing the injection of information from empty patches and focusing the limited state capacity on effective scattering regions, thereby improving the quality of visual encoding. Finally, a Hierarchical Spatial Summarization (HSS) strategy is proposed, which inserts learnable summary tokens at different depths and spatial regions of the encoder. These tokens form a visual prefix carrying multi-scale semantics and spatial bias through bidirectional state aggregation. During the text decoding stage, the summary prefix is fully provided to the decoder, enabling each generation step to directly access structured visual memory.

In summary, the main contributions of this paper are as follows.

- ISARCap 1.0, the first ISAR image captioning dataset, was constructed. It comprises 514,825 image–text pairs, including ISAR images of 38 classes of space targets and corresponding multiple natural language captions.
- ISAR-Mamba was proposed. It employs a dual-stream asymmetric visual encoder to extract complementary features, and introduces a sparsity-aware gating mechanism that leverages an energy prior-assisted scoring module to dynamically suppress empty-patch information, enabling the network to focus on effective scattering regions.
- HSS was designed. It inserts learnable summary tokens at different encoder depths and spatial groups, integrates multi-scale regional information through bidirectional state aggregation, and provides the decoder with a structured visual prefix, alleviating the information bottleneck of SSMs during generation.
- Experiments were conducted on the ISARCap 1.0 dataset, suggesting the effectiveness of the proposed method and the usability of the dataset.

The remainder of this paper is organized as follows. Section 2 briefly reviews related work. Section 3 introduces the constructed dataset, ISARCap 1.0. Section 4 details the proposed ISAR-Mamba. Section 5 presents comprehensive experimental results and analysis, demonstrating the effectiveness of the proposed method. Section 6 concludes the paper.

## 2. Related Work

### 2.1. Natural Image Captioning

Early image captioning methods [32,33,34] primarily relied on template filling or retrieval-based generation, which were constrained by fixed sentence patterns and rules, resulting in generated content lacking flexibility and diversity. With the rise of deep learning, methods based on the encoder–decoder framework gradually became mainstream, producing a series of milestone works.

Vinyals et al. [14] were the first to combine CNN with Long Short-Term Memory (LSTM), constructing an end-to-end trainable image captioning model. The model utilizes a CNN to extract global image features, and then an LSTM decodes these features into natural language sentences word by word. Subsequently, Xu et al. [35] introduced the attention mechanism into the decoding process, enabling the model to dynamically focus on different spatial regions of the image when generating each word, which significantly improved the accuracy and detail richness of the generated captions. On this basis, Anderson et al. [36] employed Faster R-CNN-based bottom-up attention to first detect salient object regions in the image and then generate captions, further improving caption quality.

To address the limitation of RNNs in modeling long-range dependencies, the Transformer architecture was introduced into the field of image captioning. Encoder–decoder models based purely on self-attention mechanisms, such as multimodal Transformers [37,38] and grid-feature Transformers [16,39], are capable of capturing global context and relationships between objects in images, and have gradually replaced LSTM as the mainstream decoder. Researchers have also proposed a series of enhanced architectures: the Key-Region Semantic Information Augmented Transformer (KSAT) [40] compensates for the inadequacy of grid features by mining semantic information from key regions in the image, and introduces an adaptive gating mechanism to dynamically adjust the alignment between image and text features; while the Generative Image-to-Text Transformer (GIT) [41] unifies visual encoding and text generation into an autoregressive task, simplifying the architectural design.

In recent years, the emergence of large-scale vision–language pretrained models has further advanced the development of image captioning. Contrastive learning models, represented by CLIP [42], have acquired strong cross-modal alignment capabilities by training on hundreds of millions of image–text pairs, making zero-shot captioning possible. Works such as BLIP [43] and CoCa [44] have introduced generative pretraining objectives on the basis of contrastive learning, achieving excellent performance in both understanding and generation tasks. More recent studies have begun to bridge frozen large language models (LLMs) with visual encoders. For instance, BLIP-2 utilizes a Q-Former to align visual and linguistic features [45], while MiniGPT-4 [46] and LLaVA [47] endow LLMs with image captioning and question-answering capabilities through instruction tuning. Such models have achieved an unprecedented level of naturalness and richness in caption generation.

### 2.2. State Space Model

SSMs originate from classical control theory and, in recent years, have received widespread attention in the field of deep learning as a new paradigm for sequence modeling. Unlike traditional RNNs and Transformers, SSMs define the continuous evolution of system states through a set of linear ordinary differential equations, which, after discretization, can efficiently process long-sequence data.

The Structured State Space Model (S4) proposed by Gu et al. [48] significantly reduces computational overhead by parameterizing the state matrix in a diagonal-plus-low-rank form, and demonstrates excellent performance in long-range dependency modeling. S4 and its subsequent variants, such as DSS [49] and S4D [50], establish SSMs as fundamental building blocks of deep networks, achieving outstanding results in tasks such as speech, text, and image classification. The core advantage of these models lies in their theoretical linear computational complexity, enabling them to efficiently process sequences over multiple time steps. However, the aforementioned SSMs are linear time-invariant systems, whose parameters do not vary with the input during inference, thereby limiting the model’s ability to dynamically select contextual information. To remedy this deficiency, Gu et al. [20] proposed Mamba, a selective SSM. Mamba integrates the selective SSM and the multilayer perceptron (MLP) module of Transformers into a unified framework, while incorporating a hardware-aware parallel scan algorithm, significantly improving training and inference efficiency. It demonstrates performance surpassing that of same-scale Transformers on tasks such as language modeling and genomic sequence analysis.

Given Mamba’s potential for long-sequence modeling with linear complexity, researchers quickly introduced it into the field of computer vision. Vision Mamba (ViM) [21] first flattens an image into a one-dimensional patch sequence and fuses contextual information from both forward and backward directions through bidirectional SSM, demonstrating better performance than ViT in image classification and segmentation tasks. VMamba [22] further designs a four-direction scanning strategy, aggregating image features from four directions—horizontal forward, horizontal backward, vertical forward, and vertical backward—effectively alleviating the spatial information loss caused by the two-dimensional non-causal structure in unidirectional SSMs. Subsequent works, such as LocalMamba [23] and EfficientVMamba [51], improve receptive field coverage and computational efficiency through local scanning windows and skip scanning paths, respectively. These methods demonstrate that, with carefully designed scanning paths, SSMs are able to learn rich structural relationships within visual data.

In multimodal and generative tasks, Mamba has also begun to emerge. Mamba-based visual encoders have been adopted as the visual backbones of multimodal large models [52,53] to reduce the computational overhead caused by quadratic attention. Some works [54,55] have attempted to unify visual encoding and text generation within the Mamba framework, leveraging the causality of SSMs to achieve efficient cross-modal autoregression.

Unlike existing Mamba encoders that adopt symmetric square patch partitioning and uniform multi-directional scanning, ISAR-Mamba handles the anisotropy of ISAR images through dual-stream asymmetric patch partitioning and scanning. Moreover, ISAR images exhibit sparsity that is absent in natural images, so SAGM is designed to enhance feature selection. In addition, some Mamba-based image captioning models encode visual features into a single visual vector, lacking differentiated spatial information modeling, whereas HSS can provide more support for text generation by preserving hierarchical spatial information.

## 3. ISARCap 1.0 Dataset

**Figure 1:**
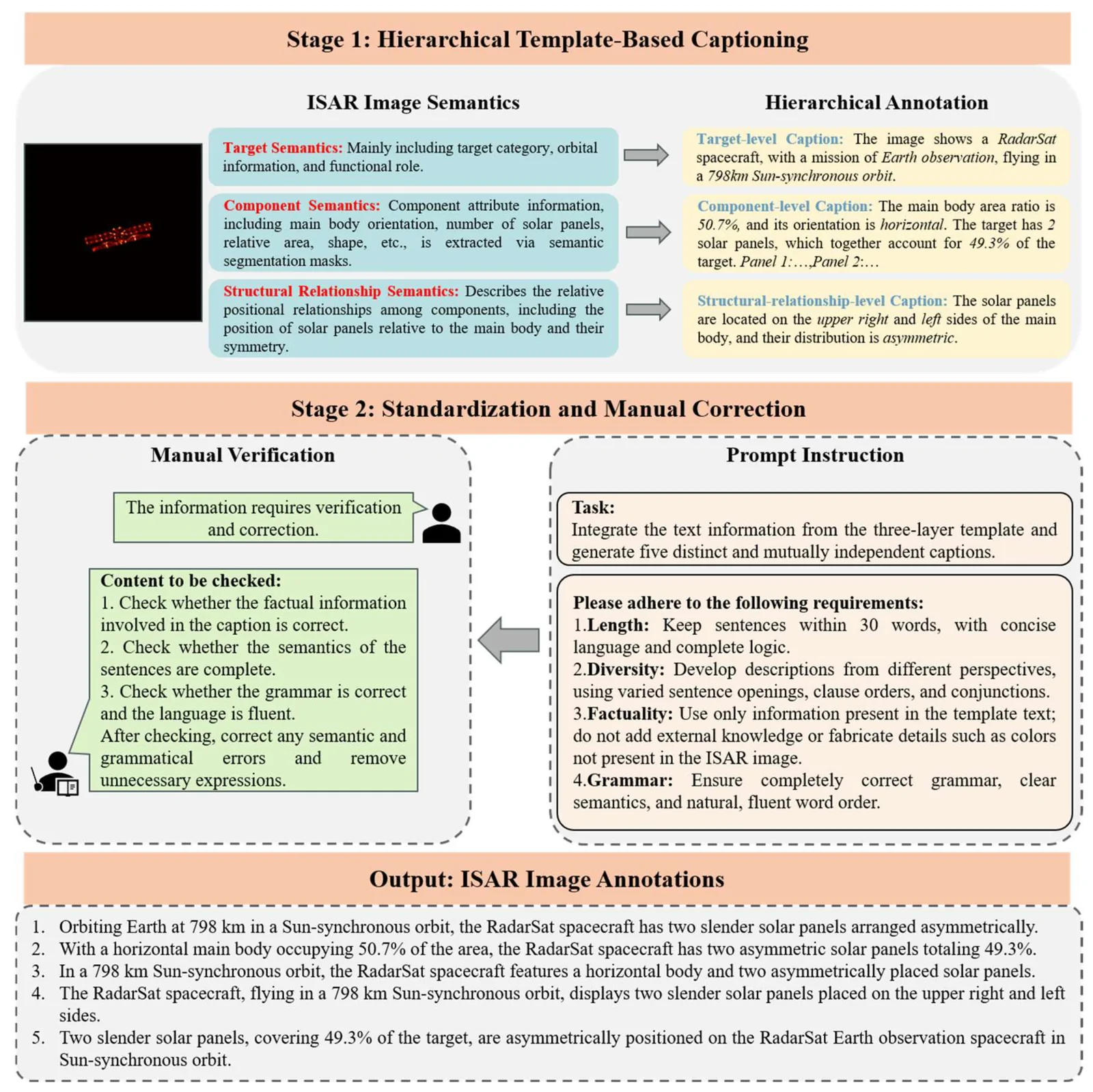
Two-stage annotation strategy: Stage 1 generates target-level, component-level and structural-relationship-level captions via hierarchical template-based representation; and Stage 2 refines the annotations through prompt standardization and manual correction.

ISARCap 1.0 is the first dataset for ISAR image captioning of space targets, designed to support the generation of fine-grained natural language captions. The dataset contains ISAR images of 38 classes of space targets, with each image accompanied by five natural language captions with varied wording. Specifically, the annotation was generated through a two-stage strategy, as shown in Figure 1. In the first stage, this paper adopted a hierarchical template-based representation method to encode semantic information at different granularities. First, target-level captions were generated based on known information. Then, to enrich the semantic information, different approaches were designed for component information extraction, thereby generating component-level and structural-relationship-level captions. This design mitigates the rigidity of traditional template-based captions while maintaining semantic accuracy. In the second stage, by applying carefully designed prompts to integrate information from all levels, these structured template texts were transformed into fluent and diverse natural language captions, thereby synthesizing five different and coherent captions for each image. Afterwards, manual review was conducted to verify factual correctness and language quality, correcting or eliminating any erroneous expressions, thereby ensuring semantic accuracy, linguistic consistency, and varied wording.

### 3.1. Hierarchical Template-Based Captioning

In the domain of natural images, image captioning typically relies on clear edge structures and salient semantic features. However, due to the unique imaging mechanism of ISAR images, there is considerable uncertainty in terms of structural integrity, target identifiability, and contextual consistency. Therefore, systematically extracting and organizing semantic information from ISAR images has become a key challenge for generating high-quality captions.

To address this challenge, this paper introduced a hierarchical template-based representation method that decomposes the visual content of space target ISAR images into three levels of semantics: target-level, component-level, and structural-relationship-level. This representation aligns with the way human experts interpret ISAR images, namely, first achieving an overall understanding of the target and then perceiving its structural components. This method decomposed the entire captioning task into three interrelated levels, each of which focused on organizing information at a specific granularity. Such hierarchical decomposition enabled systematic and fine-grained annotation while reducing subjective discrepancies. Meanwhile, to avoid overly rigid generated templates, multiple templates were designed for each level. Specifically, the annotation process was designed as follows.

(1) Target-level: The target level focused on the overall information of the target. It included the following contents: target type, operating orbit, and functional role. This information was incorporated into target-level templates to generate target-level captions, for example: “This is a {class name} spacecraft operating in {orbit}, primarily used for {function}.”

(2) Component-level: The component level focused on the attributes of the main components of space targets. Since the dataset contains multiple classes of space targets with significant structural differences between classes, the component level mainly focused on two structural elements common to all targets: the main body and the solar panels. Component attribute information was extracted through semantic segmentation masks of the space targets, as described below.

*(1) Main body orientation:* The orientation of the principal axis of the main body region, computed by performing principal component analysis (PCA) on all pixel coordinates belonging to the main body mask.

*(2) Number of solar panels:* Determined by performing connected component analysis on the solar panel masks.

*(3) Relative area of the main body:* The ratio of the number of pixels in the main body mask to the number of pixels in the entire space target mask.

*(4) Relative area of solar panels:* The ratio of the number of pixels in the solar panel masks to the number of pixels in the space target mask.

*(5) Solar panel aspect ratio:* The width-to-height ratio of the axis-aligned bounding box enclosing each connected solar panel region. This ratio characterizes whether the solar panel is square, elongated, or irregular.

The above information is incorporated into component-level templates to generate component-level captions, for example: “The main body occupies XX of the target and is oriented XX. The target has XX solar panels, which together account for XX of the target. Solar panel 1…”

(3) Structural-relationship-level: The structural relationship level modeled the relative spatial relationships between components. To enhance interpretability, the following structural spatial relationships were explicitly annotated.

*(1) Position of solar panels relative to the main body:* The centroids of each solar panel mask and the main body mask were calculated, and the quadrant was determined.

*(2) Symmetry of solar panels:* The areas of the solar panels and their distances to the main body were compared.

The above information is incorporated into structural-relationship-level templates to generate structural-relationship-level captions, for example: “The solar panels are located {positions} relative to the main body, and their distribution is {symmetry}.”

These three semantic levels together constituted a complete captioning framework, which directly supported the construction of the ISARCap 1.0 dataset.

### 3.2. Prompt Standardization and Manual Correction

In the first stage, ISAR images were annotated from three perspectives: target-level, component-level, and structural-relationship-level. However, although these templates were complete and accurate as described above, the sentences generated by templates were essentially fixed and repetitive, lacking lexical diversity, syntactic variation, and natural fluency. Meanwhile, when providing a complete caption for an ISAR image, a fixed set of templates could cover all effective information across all levels, resulting in stiff wording, redundant expressions, or unnatural transitions between different information levels.

To bridge the gap between structured templates and natural language captions, this work further introduced a prompt standardization stage. The purpose of this stage was to synergize the structured knowledge of templates with the generation capabilities of LLMs, thereby improving the scientific rigor of annotation while ensuring sentence quality and structural consistency. The design of structured prompts played a pivotal role, as it directly determined the LLM’s ability to extract and synthesize template information. Subsequently, carefully designed prompt instructions were used to synthesize all template information components, guiding the generation of five differently expressed natural language captions for each image. At this stage, the LLM was not used to directly interpret ISAR images, nor was it allowed to fabricate new semantic content beyond the provided template information. It was only used to fuse the three layers of pre-extracted template facts and generate multiple new complete captions. This restriction ensured that the factual completeness established by the hierarchical template representation was preserved, while greatly enriching the forms of captions. The information about the LLM used was as follows: the version was DeepSeek-V4-Pro [56], with a temperature of 0.9 and a top_p of 0.95. The main prompt is as follows.

(1) Length: Keep sentences within 30 words, with concise language and complete logic.

(2) Diversity: Develop descriptions from different perspectives, using varied sentence openings, clause orders, and conjunctions.

(3) Factuality: Use only information present in the template text; do not add external knowledge or fabricate details such as colors not present in the ISAR image.

(4) Grammar: Ensure completely correct grammar, clear semantics, and natural, fluent word order.

After the LLM generated candidate captions, a manual correction process was introduced. Manual correction was performed by three master’s students in this field, who have several years of experience in radar image interpretation, ensuring the correctness of the interpretation. During manual correction, annotations that did not meet the requirements were revised to meet the requirements. When uncertainty arose during correction, a professor working in this field made the final decision. Rejected captions were not removed from the dataset; instead, each rejected caption was regenerated and re-reviewed until it passed the criteria. Consequently, every image retained five captions, and the number of final annotations equaled the number of original annotations; no sample was permanently discarded at any stage. In addition, 10% of all descriptions were randomly selected for double review to control annotation quality, and the Cohen’s kappa on the double-reviewed subset was 0.91. The correction process followed three criteria: factual correctness, semantic completeness, and linguistic fluency. Human annotators carefully inspected the automatically generated captions, correcting or eliminating any erroneous expressions to ensure semantic completeness and accuracy. The text captions that passed the review were retained as the corpus of the ISARCap 1.0 dataset for model training and validation. After manual review, the quantitative quality statistics were as follows: 87.6% (450,987) of the annotations were accepted directly without modification, 9.8% (50,452) were corrected, and 2.6% (13,386) were rejected and regenerated.

This stage transformed fixed templates into natural and diverse language without altering the factual content. Meanwhile, manual correction ensured that the text content conformed to the dataset construction standards. This hybrid approach effectively balanced automation efficiency and annotation quality.

### 3.3. ISARCap 1.0 Dataset Analysis

Based on the two-stage annotation strategy, ISARCap 1.0, the first structured ISAR image captioning dataset for space targets, was constructed. This dataset aims to provide high-quality semantic support for language modeling on space target ISAR images and alleviate the long-standing problem of the lack of aligned image–text resources in the ISAR domain.

ISARCap 1.0 employs ISAR images of 38 different classes of space targets, with each class imaged under multiple attitudes. The simulation parameters were set as follows: center frequency of 17 GHz, bandwidth of 2 GHz, ISAR image resolution of 0.075 m, and image size of 512 × 512. The radar and target were modeled based on a turntable model, and after obtaining the radar echoes, the Range-Doppler algorithm was used for ISAR imaging.

**Figure 2:**
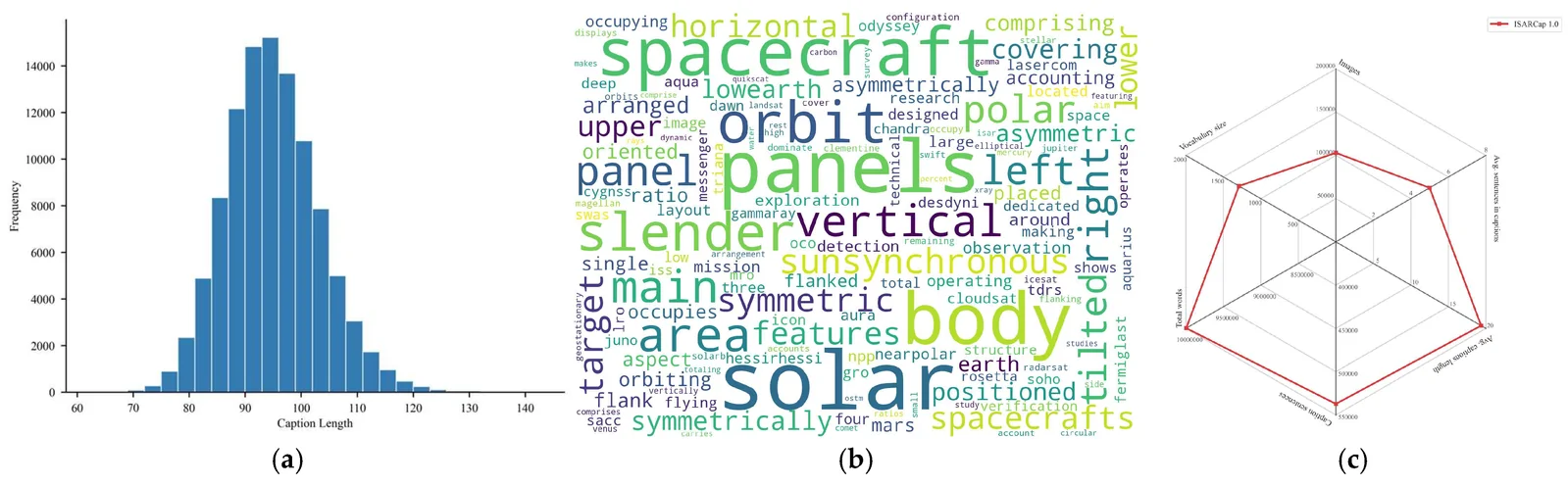
Statistics of the ISARCap 1.0 dataset. (**a**) Frequency histogram of the total caption length per image—frequency distribution. (**b**) Word cloud image of the primary annotation words in the dataset—word cloud. (**c**) Radar plot of the main data in the ISARCap 1.0 dataset.

As shown in Figure 2, a comprehensive dataset analysis was conducted to further characterize the linguistic properties of ISARCap 1.0, reflecting its richness and task alignment. ISARCap 1.0 includes 102,965 images and 514,825 image–text pairs, comprising a total of 9,971,695 words, with an average sentence length of 19.4 words. As shown in Figure 2a, most sentences contain 16 to 22 words, exhibiting a concise and clear sentence structure that conforms to standard language norms and also reflects the richness of the annotated content. The word cloud in Figure 2b highlights high-frequency terms such as “spacecraft,” “solar panels,” and “main body,” indicating that the annotated semantics are highly relevant to the ISAR domain. In addition, Figure 2c systematically summarizes statistics from multiple dimensions, including image count, annotation density, and vocabulary coverage, further validating the scale and semantic coverage of the dataset.

The design of ISARCap 1.0 integrates the imaging characteristics and cognitive structure of ISAR images, and mainly exhibits three advantages. First, the samples cover multiple target classes and attitudes, demonstrating significant intra-class differences, which provides support for model generalization. Second, each image is associated with multi-level, multi-view, and multi-style linguistic expressions, facilitating more fine-grained image captioning and reasoning tasks. Finally, based on unified templates and structured rules, the dataset can be extended to other ISAR target types. ISARCap 1.0 is the first image–text aligned dataset in the ISAR domain to date. Its structured semantic organization and high-quality annotations provide clear semantic supervision and explicit guidance for subsequent image-language modeling tasks.

## 4. Methodology

### 4.1. Architecture Overview

In recent years, deep neural networks have demonstrated great potential in image captioning tasks, providing a new paradigm for the automatic interpretation of space target ISAR images. However, the inherent sparsity and physical dimensional anisotropy of ISAR images make it difficult for existing vision models to be directly transferred to the ISAR image captioning task, constraining the development of this field.

To address the above problems, this paper proposes ISAR-Mamba, a dual-stream gated SSM for ISAR image captioning. Its core idea is to adapt the imaging characteristics of the two physical dimensions of ISAR images through dual-stream asymmetric encoding, dynamically suppress the interference of empty patches on hidden states through a sparsity-aware gating mechanism, and inject multi-scale, spatially structured visual memory into the text decoder in the form of a prefix through a hierarchical spatial summarization strategy, so that each step of the text generation process can perceive visual information at different levels.

**Figure 3:**
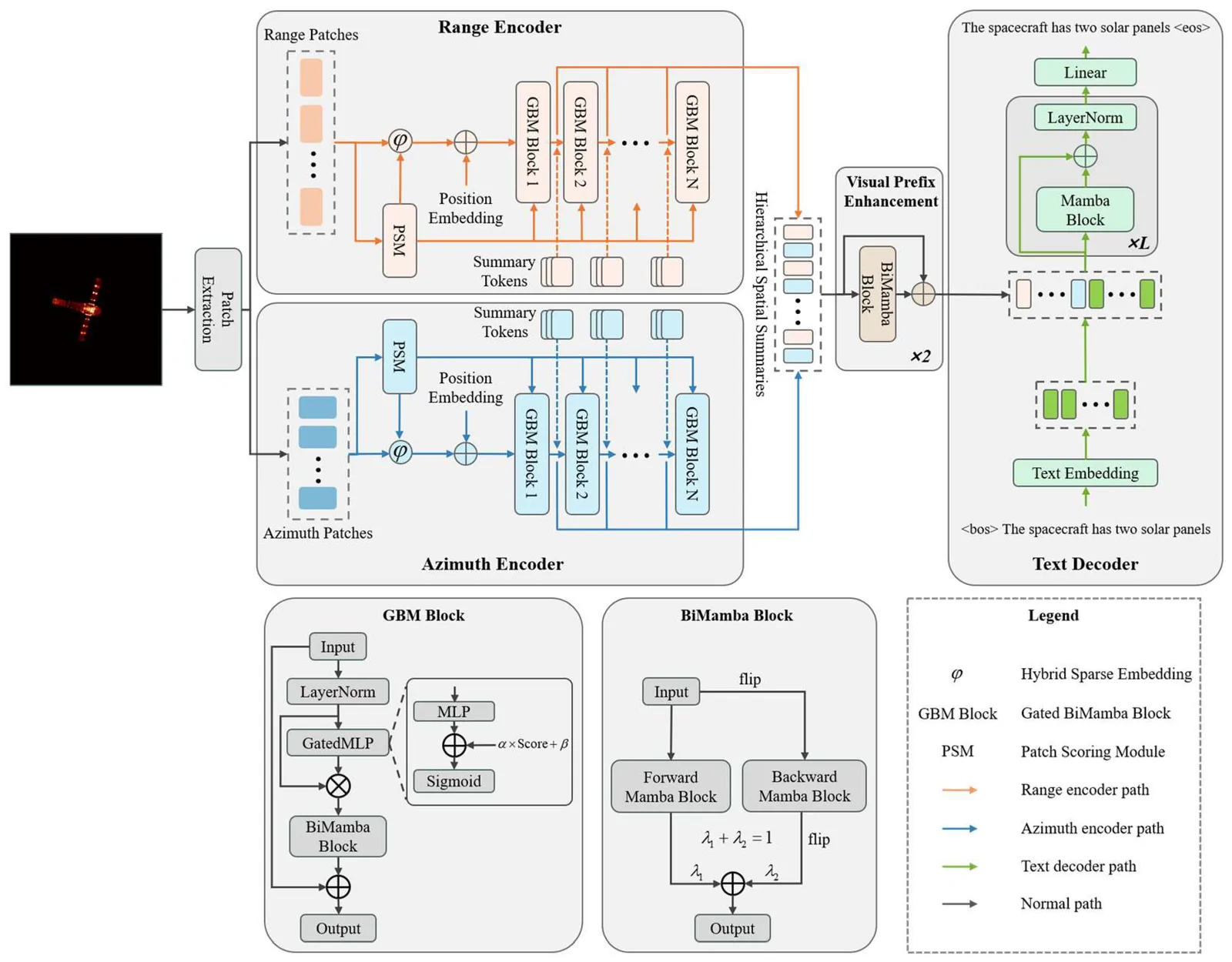
Overall structure of ISAR-Mamba. The model first partitions the input into range patches and azimuth patches and feeds them into two parallel branches, which perform visual feature extraction according to their respective scanning strategies. The sparsity-aware gating mechanism is applied therein to regulate information flow, and summary tokens are inserted to obtain the visual prefix. Subsequently, visual prefix enhancement is performed, and the result enters the text decoder to generate text captions.

The overall architecture of ISAR-Mamba is shown in Figure 3, which mainly consists of four parts. DSAE partitions the input ISAR image into asymmetric patches along the range and azimuth dimensions, respectively, to capture the macroscopic contour and scattering details of the target, enabling the SSM sequences to extract visual features simultaneously along two complementary dimensions. SAGM first extracts scattering point energy from patches as a physical prior. It then combines this prior with a learnable scoring module to generate importance scores. Through differentiable gating, SAGM modulates the input of the SSM and concentrates the limited state capacity on effective scattering regions. HSS inserts learnable summary tokens at specified intermediate layers and spatial groups of the encoder. Through bidirectional SSM aggregation, these tokens form a visual prefix carrying multi-scale semantics and spatial bias, enabling the subsequent decoder to directly access structured visual memory at each generation step. The text decoder is mainly composed of multiple stacked one-dimensional Mamba layers. It only receives the hierarchical summary tokens as the visual prefix and generates image captions in an autoregressive manner.

### 4.2. Dual-Stream Asymmetric Encoder (DSAE)

The physical characteristics of ISAR images determine that there are essential differences between the range and azimuth dimensions in terms of scattering mechanisms, target representations, and information density. Existing Mamba-based vision methods typically adopt square patches and symmetric multi-directional scanning paths, which fail to perform differentiated modeling for this anisotropy.

To address this issue, the DSAE is designed. Its core idea is to construct two parallel and structurally complementary sequence modeling paths along the range and azimuth dimensions of ISAR images, respectively. Through asymmetric patch partitioning and scanning strategies, the two streams are enabled to focus on the primary information characteristics of their respective dimensions, thereby providing richer and more physically meaningful visual representations for subsequent text decoding.

**Figure 4:**
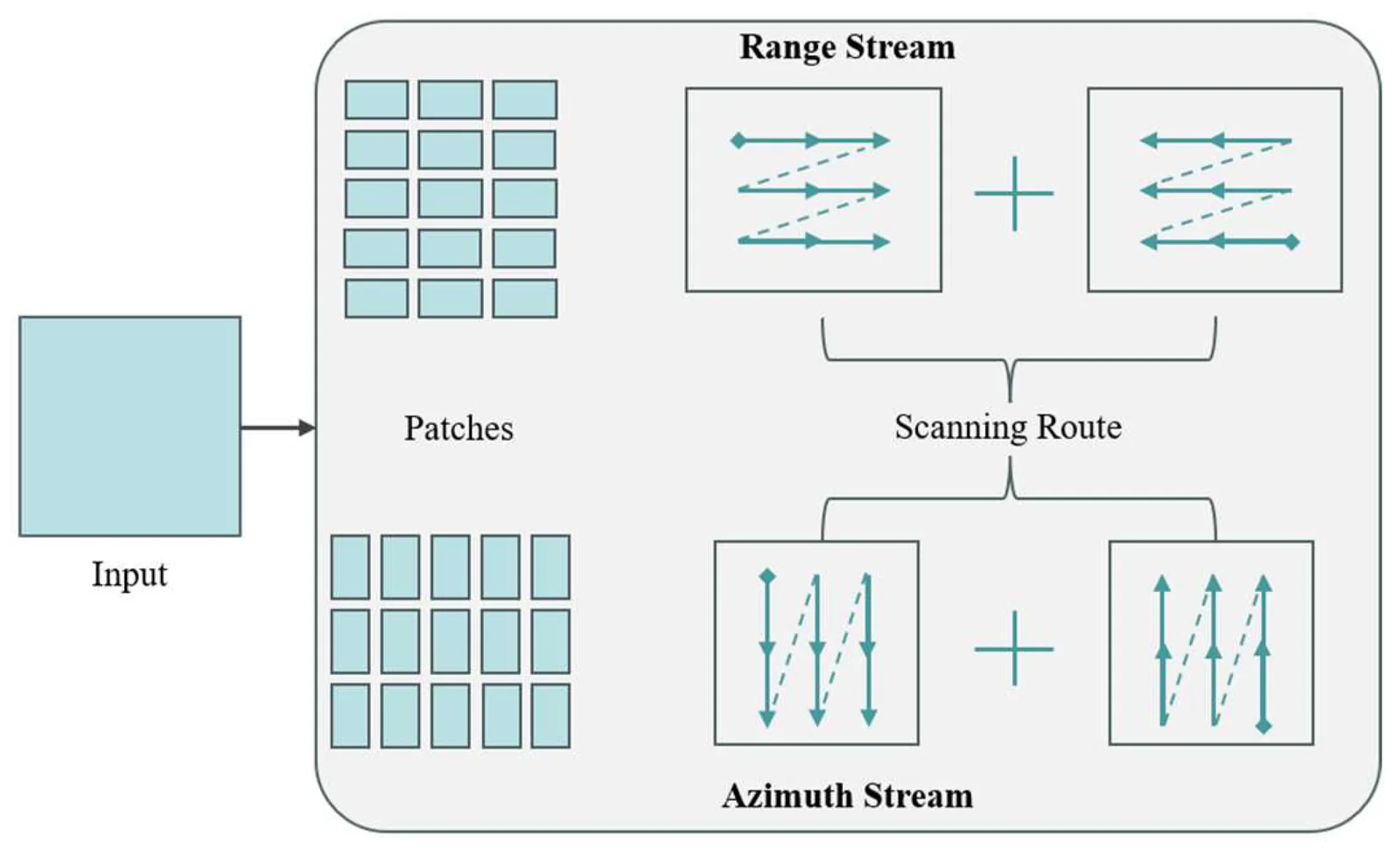
Patch partitioning and scanning strategy.

Let the input ISAR image be denoted as $I\in {\mathbb{R}}^{H\times W}$, where H represents the number of azimuth sampling points and W represents the number of range sampling points. In this paper, H=W=512. The dual-stream patch partitioning and scanning strategy is illustrated in Figure 4.

The range stream adopts a larger range-dimension patch size $p_{w}^{R}$ and a smaller azimuth-dimension patch size $p_{h}^{R}$ ($p_{w}^{R}>p_{h}^{R}$), so that each patch covers a wider radial region along the range dimension, which facilitates capturing the macroscopic contour of the target. The image is partitioned into a grid of $H_{R}=H/p_{h}^{R}$ rows and $W_{R}=W/p_{w}^{R}$ columns, yielding a total of $N_{R}=H_{R}\times W_{R}$ image patches. To strengthen the long-range dependency along the range dimension, this stream adopts a range-wise scanning strategy: first, an azimuth index $i\in [1,H_{R}]$ is fixed, and then all $W_{R}$ patches in that row are arranged sequentially along the range dimension. The final sequence is obtained by traversing all rows. Therefore, the k-th token in the sequence corresponds to the coordinate (i,j) in the original grid, which satisfies the following formula. This arrangement ensures that adjacent tokens are continuous along the range dimension, enabling the subsequent SSM to accumulate information along this direction.

(1) $$k=(i-1)\cdot W_{R}+j,\;\;\;i\in [1,H_{R}],j\in [1,W_{R}]$$

The azimuth stream, in contrast, employs a larger azimuth-dimension patch size $p_{h}^{A}$ and a smaller range-dimension patch size $p_{w}^{A}$ ($p_{h}^{A}>p_{w}^{A}$), so as to highlight scattering details along the azimuth direction. The corresponding grid dimensions are $H_{A}=H/p_{h}^{A}$ and $W_{A}=W/p_{w}^{A}$, with a total number of tokens $N_{A}=H_{A}\times W_{A}$. This stream adopts an azimuth-wise scanning strategy: first, a range index $j\in [1,W_{A}]$ is fixed, and then all $H_{A}$ patches in that column are arranged sequentially along the azimuth direction. The final sequence is obtained by traversing all columns. Therefore, the token order satisfies

(2) $$k=(j-1)\cdot H_{A}+i,\;\;\;i\in [1,H_{A}],j\in [1,W_{A}]$$

For each patch, a shared linear projection is used to map its flattened vector into a d-dimensional embedding:

(3) $$v_{i,j}=W_{p}\cdot \mathrm{flatten}(I_{i,j})+b_{p}$$

where $W_{p}\in {\mathbb{R}}^{(p_{h}p_{w})\times d}$ and $b_{p}\in {\mathbb{R}}^{d}$ are learnable parameters. The range stream and the azimuth stream perform the above projection with their respective patch sizes to generate their corresponding token sequences $V^{R}\in {\mathbb{R}}^{N_{R}\times d}$ and $V^{A}\in {\mathbb{R}}^{N_{A}\times d}$.

Flattening the grid into a 1D sequence inevitably loses spatial position information. To this end, a learnable 2D positional encoding is independently maintained for each stream. For the range stream, position embedding parameters $P^{R}\in {\mathbb{R}}^{H_{R}\times W_{R}\times d}$ are defined, the corresponding vector is retrieved according to the token’s grid coordinates (i,j), and added to the patch embedding:

(4) $${\tilde{v}}_{i,j}^{R}=v_{i,j}^{R}+P_{i,j}^{R}$$

The azimuth stream similarly possesses its own independent positional encoding $P^{A}\in {\mathbb{R}}^{H_{A}\times W_{A}\times d}$, yielding the enhanced token representation ${\tilde{V}}^{A}$. This explicit injection of positional information enables the model to distinguish tokens from different regions based on their coordinate encodings, thereby compensating for the deficiency of SSMs in 2D spatial perception.

As shown in Figure 3, each stream is composed of multiple stacked encoding blocks with identical structures. The basic unit of an encoding block is the bidirectional Mamba module, which aggregates global context from both directions for each token by simultaneously performing forward and reverse scans. Let the input to the current block be denoted as $X\in {\mathbb{R}}^{N\times d}$ (for simplicity, the stream identifier is omitted), and the forward branch employs a standard Mamba layer:

(5) $$F_{\mathrm{fwd}}={\mathrm{Mamba}}_{\mathrm{fwd}}(X)$$

The reverse branch first flips the input sequence along the length dimension, processes it through another independent Mamba layer, and then flips it back to restore the original order:

(6) $$F_{\mathrm{rev}}=\mathrm{flip}({\mathrm{Mamba}}_{\mathrm{bwd}}(\mathrm{flip}(X)))$$

The outputs of the two paths are fused through a learnable gating mechanism, enabling the model to dynamically adjust the context from different directions:

(7) $$F_{\mathrm{out}}=\lambda ⊙F_{\mathrm{fwd}}+(1-\lambda )⊙F_{\mathrm{rev}}$$

where λ is a learnable parameter and ⊙ denotes the Hadamard product.

### 4.3. Sparsity-Aware Gating Mechanism (SAGM)

The sparsity of ISAR images makes the large number of background patches generated by regular uniform partitioning an important factor affecting the quality of SSM encoding. If all patches are injected into the state space with equal importance without distinction, the redundant information carried by empty patches will continuously dilute the model’s focus on effective scattering information, degrading the quality of feature representations.

To address this problem, SAGM is designed. It suppresses invalid information from two levels, namely patch embedding and SSM state update, guiding the model to concentrate its limited state capacity on effective scattering regions.

**Figure 5:**
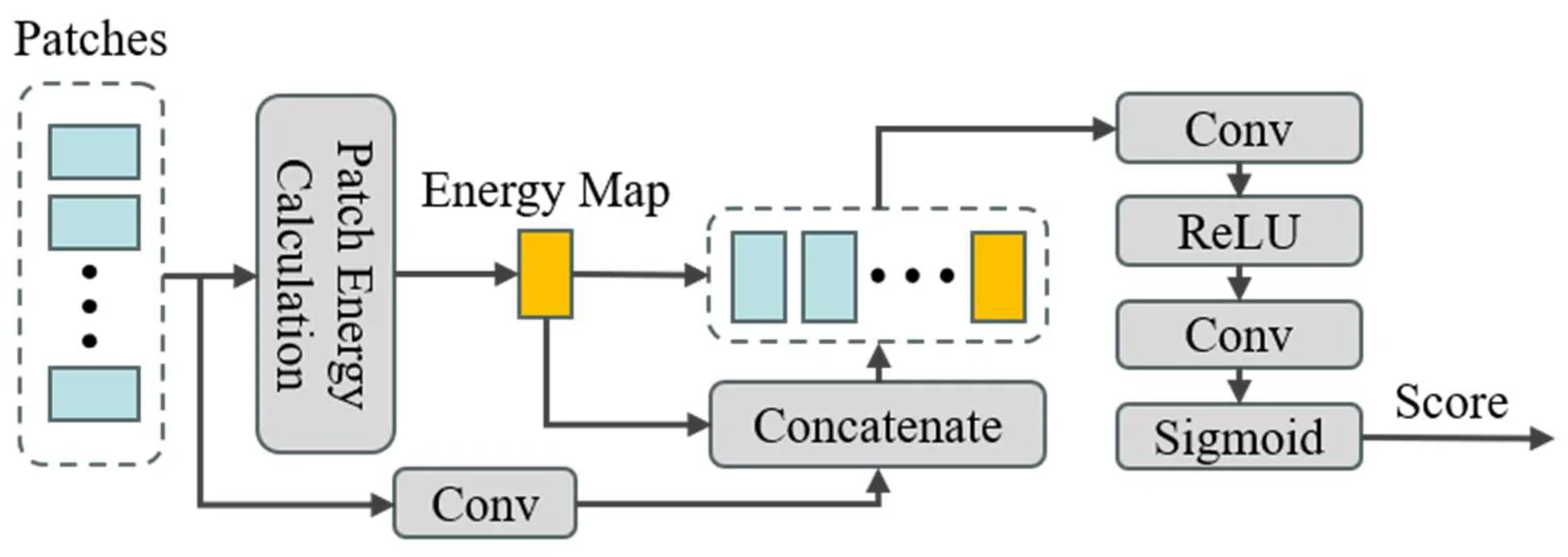
Structure of the PSM. First, the patch energy map is computed, and then it is combined with the learnable feature extraction module to jointly generate the importance scores.

SAGM first generates an importance score for each patch through a Patch Scoring Module (PSM), where the score is jointly determined by the patch energy prior and learnable features. The structure of the PSM is shown in Figure 5. Considering that the energy intensity of scattering points in an ISAR image directly reflects the attributes of the target, the normalized energy map $E\in {\mathbb{R}}^{H_{p}\times W_{p}}$ of each patch is first computed as:

(8) $$E_{i,j}=\frac{\|I_{i,j}{\|}_{2}-\min\|I{\|}_{2}}{\max\|I{\|}_{2}-\min\|I{\|}_{2}+\epsilon }$$

where $I_{i,j}$ denotes the (i,j)-th patch, $\|\cdot {\|}_{2}$ is the L2 norm, and ε is a tiny constant to prevent division by zero.

Subsequently, the feature maps obtained by passing all image patches through a convolutional layer are concatenated with the energy map along the channel dimension and fed into the scoring module:

(9) $$s_{i,j}=\sigma \left({\mathrm{Conv}}_{2}\left(\mathrm{ReLU}\left({\mathrm{Conv}}_{1}\left(\left[{\mathrm{Conv}}_{0}(I);E\right]\right)\right)\right)\right)$$

where σ is the sigmoid function.

After the importance score s is obtained, a soft mixing operation is applied to the patch embeddings to adaptively replace the representations of empty patches. Given the original patch embedding $v^{\mathrm{raw}}$ (the embedding introduced in Section 4.2), a globally learnable empty-patch placeholder $e_{\mathrm{empty}}\in {\mathbb{R}}^{d}$ is introduced. The final mixed embedding is then formulated as:

(10) $$v=s\cdot v^{\mathrm{raw}}+(1-s)\cdot e_{\mathrm{empty}}$$

When s→0, the token degenerates into a unified empty placeholder; when s→1, the original embedding is retained. This soft replacement not only suppresses the information from empty patches but also maintains gradient stability.

Although the mixed embedding suppresses the information from empty patches at the initial encoding stage, subsequent SSM layers may reactivate these tokens during state interaction and affect the global representation through cross-layer propagation. Therefore, a token-wise state update gating mechanism is further introduced in each bidirectional Mamba encoding block, as shown in Figure 3. Specifically, let the input sequence of the l-th layer be $X^{\left(l\right)}$, and the corresponding score sequence be s. Layer normalization is first applied as:

(11) $$X_{norm}^{\left(l\right)}=\mathrm{LayerNorm}(X^{\left(l\right)})$$

Then, a content-adaptive gating coefficient $g\in {\mathbb{R}}^{N\times 1}$ is generated as:

(12) $$g=\sigma (\alpha \cdot s+\beta +\mathrm{MLP}(X_{norm}^{\left(l\right)}))$$

where α and β are learnable parameters.

This gating coefficient integrates the sparsity prior s, jointly estimated from energy and image content, the global bias β, and the contextual features of the current token, thereby endowing the gating mechanism with content-adaptive capability. Finally, the gating operation is applied to the normalized input:

(13) $$X_{\mathrm{gated}}^{\left(l\right)}=g⊙X_{norm}^{\left(l\right)}$$

The sequence filtered by the gating operation is then fed into the bidirectional Mamba module to complete the state update.

Through the dual constraints of the mixed embedding and the state-update gating, SAGM enables the SSM encoder to adaptively allocate limited state capacity to regions with physical scattering significance without introducing attention or requiring manual thresholds, thereby improving the quality of visual encoding.

### 4.4. Hierarchical Spatial Summarization (HSS)

Some existing Mamba-based generative methods compress the visual features of the encoder into a single context vector and feed it to the text decoder, which makes it difficult to effectively utilize visual information at different granularities. In contrast, if the entire visual feature sequence is fed to the decoder as a prefix, not only does the long sequence increase the burden of SSM state storage, but early visual information also tends to be neglected by subsequent text tokens during autoregressive generation, making it difficult to continuously provide fine-grained visual information.

To address this problem, the HSS strategy is proposed. It extracts structured visual prefixes from different depths and different spatial regions of the encoder without introducing any attention mechanism. Its core idea is as follows: at several intermediate layers of the encoder, the current visual token sequence is equally divided into K groups according to spatial regions, and a learnable local summary token is inserted at the end of each group. Through bidirectional SSMs, these tokens interact with both the tokens within their own group and global tokens, thereby aggregating summary representations that contain both the local context of the region and global information. By repeating this process at different depths, multi-level and structured spatial memories can be obtained. Finally, the summary tokens from all layers and all streams are collected and arranged into a concise visual prefix, which is directly accessible to the decoder at every generation step.

(1) Spatial grouping and summary token insertion. Let the input sequence of a certain encoding stream at the l-th layer be $X^{\left(l\right)}\in {\mathbb{R}}^{N\times d}$. As described in Section 4.2, the ordering of this sequence corresponds to row-major or column-major scanning of the image, and thus the positions within the sequence implicitly encode the two-dimensional spatial coordinates of the image. $X^{\left(l\right)}$ is equally divided into K groups along the sequence dimension:

(14) $$X^{\left(l\right)}=[X_{1}^{\left(l\right)};X_{2}^{\left(l\right)};\ldots ;X_{K}^{\left(l\right)}],\;\;\;X_{K}^{\left(l\right)}\in {\mathbb{R}}^{M\times d}$$

where M=N/K is the number of tokens contained in each group, and each group $X_{K}^{\left(l\right)}$ corresponds to a continuous spatial region in the image.

For this layer, a set of learnable local summary tokens $C^{\left(l\right)}=\{c_{1}^{\left(l\right)},\ldots ,c_{K}^{\left(l\right)}\}$ is maintained, where each $c_{K}^{\left(l\right)}\in {\mathbb{R}}^{d}$ is a randomly initialized vector. To distinguish them from ordinary visual tokens, a shared summary positional encoding $P_{\mathrm{sum}}\in {\mathbb{R}}^{d}$ is additionally added to all summary tokens, yielding the initial summary representation ${\tilde{c}}_{k}^{\left(l\right)}=c_{k}^{\left(l\right)}+p_{\mathrm{sum}}$. Subsequently, each ${\tilde{c}}_{k}^{\left(l\right)}$ is inserted at the end of its corresponding group, forming the extended sequence ${\tilde{X}}^{\left(l\right)}\in {\mathbb{R}}^{(N+K)\times d}$:

(15) $${\tilde{X}}^{\left(l\right)}=\mathrm{Concat}\left(\left[X_{1}^{\left(l\right)};{\tilde{c}}_{1}^{\left(l\right)}],[X_{2}^{\left(l\right)};{\tilde{c}}_{2}^{\left(l\right)}],\ldots ,[X_{K}^{\left(l\right)};{\tilde{c}}_{K}^{\left(l\right)}\right]\right)$$

(2) Bidirectional state aggregation and summary extraction. The extended sequence ${\tilde{X}}^{\left(l\right)}$ is then passed through the bidirectional gated Mamba module of this layer to obtain the output ${\tilde{Y}}^{\left(l\right)}$. Owing to the bidirectional scanning mechanism of Mamba, each summary token aggregates information from all preceding visual tokens along the forward path and absorbs the context of subsequent tokens along the backward path. This bidirectional interaction ensures that summary tokens can not only consolidate local features within their own group region but also capture global relationships across regions.

After completing the SSM computation for this layer, the output vectors at the positions corresponding to all summary tokens are extracted from ${\tilde{Y}}^{\left(l\right)}$ as the local summary features generated by this layer:

(16) $$s_{k}^{\left(l\right)}={\tilde{Y}}^{\left(l\right)}[:,{\mathrm{pos}}_{k},:],\;\;\;k=1,\ldots ,K$$

where ${\mathrm{pos}}_{k}$ is the position index of the k-th summary token in the extended sequence.

(3) Hierarchical visual prefix construction. The above process is performed independently at each layer in the selected layer set L of the encoder. For the range stream, the local summary features $S_{l}^{R}\in {\mathbb{R}}^{K\times d}\;\;\;(l\in L)$ produced at each selected layer are collected; for the azimuth stream, $S_{l}^{A}$ is obtained in the same manner. Finally, all summary features are combined in layer order into a unified visual prefix P:

(17) $$P=[S_{l_{1}}^{R},S_{l_{1}}^{A},S_{l_{2}}^{R},S_{l_{2}}^{A},\ldots ]$$

where $l_{1},l_{2},\ldots$ is the layer index in L arranged in increasing order of network depth, and the dimension of P is (2⋅|L|⋅K)×d.

(4) Fusion in the decoding stage. First, a bidirectional Mamba module is employed to perform enhanced interaction within the prefix, allowing summaries from different levels and different regions to further fuse contextual information. The enhanced prefix is then concatenated with text tokens, and the captions are generated autoregressively through multiple layers of 1D Mamba. Benefiting from the explicitly retained spatial grouping and hierarchical summaries in the prefix, the decoder is able to directly and stably access multi-level visual information when generating each word.

In summary, HSS leverages the aggregation capability of bidirectional SSMs and sequence spatial position encoding to achieve structured visual summaries, which not only avoids the memory decay caused by long sequences but also overcomes the information loss resulting from global vector compression.

## 5. Results and Discussion

In this section, a series of experiments are designed to demonstrate the effectiveness of the proposed method. Section 5.1 details the experimental settings. Section 5.2 specifies the evaluation metrics. Section 5.3 compares the proposed method with representative algorithms of the same category. On this basis, Section 5.4 conducts efficiency comparisons. Section 5.5 provides an in-depth analysis and validation of the effectiveness of each key module through ablation experiments. Section 5.6 presents further analysis and discussion. Section 5.7 presents noise robustness experiments on typical targets.

### 5.1. Experimental Setting

(1) Experimental environment and hyperparameters: All experiments in this paper were conducted on a unified hardware platform to ensure comparability of results. Specifically, a computer server equipped with two NVIDIA GeForce RTX 4090 GPUs was used. The software environment was built on PyTorch 2.7.0 with CUDA 12.6, using Python 3.10 as the programming language. The random seed was fixed to 42 in all experiments to ensure reproducibility. Throughout the training and testing process, model weights were saved and used with FP32 precision. The model was trained using the AdamW optimizer with an initial learning rate of 1 × 10^−4^, a weight decay coefficient of 1 × 10^−2^, and a cosine annealing learning rate scheduler. The cross-entropy loss function was employed during model training. The model was trained for a total of 100 epochs with a batch size of 16.

(2) Model parameters: In ISAR-Mamba, the number of encoder layers was set to 24, the number of decoder layers was set to 6, and the token encoding dimension was set to 256. For the Mamba module, the expand operator was set to 2, the hidden state dimension was 16, and the size of the causal Conv1D kernel was 4. The patch size of the range stream was 32 × 8, and the patch size of the azimuth stream was 8 × 32. The number of spatial groups was set to 8, and the number of encoder layers for summary insertion was 4.

(3) Dataset: All experiments were conducted on the ISARCap 1.0 dataset, which was split into training and test sets with a ratio of 8:2. All spacecraft shared the same simulation parameters. However, due to differences in their imaging attitude, different attitude ranges and sampling intervals were adopted for different spacecraft. Each image in ISARCap 1.0 was generated by an independent echo simulation: for every discrete attitude sample (pitch, roll, yaw), a complete radar echo was synthesized under the turntable model and was imaged individually via the Range-Doppler algorithm, so that consecutive samples did not share any coherent processing interval. The test set was formed by equal-interval sampling within the attitude sequence of each class; consequently, every test image was separated from its nearest training images by at least one full sampling interval along each attitude angle, and no test image shared its attitude with any training image. No epoch or checkpoint selection was performed on any partition. The test partition was never used for model selection, early stopping, or hyperparameter tuning, so a separate validation partition was not required under this fixed-schedule protocol.

### 5.2. Evaluation Metrics

To evaluate the performance of the proposed method and the quality of the generated sentences, eight metrics were used, including BLEU-*n* (*n* = 1, 2, 3, 4), recall-oriented understudy for gisting evaluation (ROUGE_L), METEOR, consensus-based image description evaluation (CIDEr), and S_m_.

(1) BLEU-*n* (B-*n*): Bilingual evaluation understudy (BLEU) [57] is a metric first used to evaluate the performance of machine translation methods. It measures the co-occurrences of n-grams in generated sentences and ground truth sentences. Commonly used values of *n* are 1, 2, 3, and 4.

(2) METEOR (M): Metric for evaluation of translation with explicit ordering (METEOR) is used to evaluate the accuracy of machine translation [58]. The metric is calculated by generating an alignment between the generated sentences and ground truth sentences. Unlike BLEU-1, METEOR takes into account the unigram precision and the unigram recall.

(3) ROUGE_L(R): It is a metric for evaluating automatic summarization and machine translation [59]. ROUGE_L is the F-measure of the longest common subsequence between the generated sentences and the ground truth sentences.

(4) CIDEr(C): It is specially designed for image captioning tasks [60]. It applies term frequency-inverse document frequency (TF-IDF) weights to n-grams in the generated and ground truth sentences.

(5) S_m_ [61,62,63,64] is calculated as the arithmetic mean of BLEU-4, METEOR, ROUGE_L, and CIDEr metrics. It can evaluate the quality of generated captions comprehensively.

### 5.3. Comparative Experiments

To objectively evaluate the proposed method, ISAR-Mamba was compared with 13 image captioning methods based on CNN-RNN frameworks, Transformer frameworks, and Mamba frameworks. These methods are built upon either a single architecture or a fusion of multiple architectures, covering the mainstream technical routes in the field of image captioning, thereby constituting a comprehensive and challenging comparison benchmark.

(1) Adaptive-Attention model [65] with a visual sentinel via a sentinel gate automatically decides whether to attend to image regions or rely on its language model at each decoding step.

(2) AoANet [66] introduces the Attention on Attention (AoA) module to perform a secondary filtering of attention results in both the encoder and decoder, filtering out irrelevant or misleading information to more accurately model the relevance between image regions and queries and thereby improve caption generation quality.

(3) CaptionNet [67] reduces dependence on previous predicted words by feeding only attended image features into its memory cell and initializes the memory with Image Feature Encoding (IFE), thus forcing the model to focus more on visual cues for generating image descriptions.

(4) Word-Sentence [68] framework first extracts valuable words from the image via a CNN-based multi-label classifier, and then organizes them into a well-formed sentence using a transformer-based sequence generator.

(5) MC-Net [69] aggregates multi-scale contextual information via a multi-scale feature extraction and fusion module, and employs an adaptive visual-text alignment decoder to generate accurate and fluent captions for images.

(6) PKG-Transformer [61] leverages scene-level and object-level features enhanced by graph attention networks, and incorporates a prior scene–object knowledge matrix into the transformer attention mechanism to generate more accurate and contextually relevant image captions.

(7) HCNet [62] employs hierarchical feature aggregation to comprehensively fuse multi-scale visual features and leverages cross-modal feature interaction with an alignment loss to bridge image–text modality gaps, thereby generating accurate and complete image captions.

(8) ICNET [70] extracts multiscale local features via CNN and global features via ViT, projects local features into high level semantic concepts through a concept mapping network, enhances global representations with a feature enhancement module, and employs a concept interaction module in the transformer to align visual concepts with word embeddings for accurate category and relationship captioning.

(9) Mamba-Caption [71] leverages a Mamba-based decoder with selective state space modeling to efficiently generate accurate and semantically coherent image captions in linear time.

(10) RSIC-GMamba [63] integrates genetic operations and a self-attention mechanism into the SSM to effectively capture the contextual dependencies of 2D images, and fuses multi-level visual-semantic information via a vision-scene text aggregation module to generate accurate captions.

(11) DPPF [72] integrates scene-level semantic priors into the decoder input via a pre-fusion mechanism while simultaneously leveraging CLIP visual grid features for cross-attention, enabling early semantic conditioning and fine-grained spatial grounding to generate coherent and accurate captions.

(12) CSSA [73] achieves cross-modal spatial–semantic alignment via a multi-branch contrastive learning mechanism and a dynamic geometry Transformer that incorporates spatial geometry information, thereby generating more accurate and coherent image captions.

(13) CDRE [64] fine-tunes the CLIP image encoder for semantic alignment, dynamically fuses global and local features via a gated modulation module, and incorporates explicit spatial relation modeling through relation enhanced attention to generate more accurate and semantically rich captions.

DPPF, ICNET, and CDRE employ the CLIP pretrained model in part of their modules, rather than as the whole model. In these three methods, only the modules that depend on the CLIP representation are initialized from the released pretrained weights and frozen; all remaining trainable parts of the model, including the fusion and interaction modules and the decoder, were trained on ISARCap 1.0 under exactly the experimental setting of Section 5.1, with the same optimizer, learning-rate schedule, epoch count and batch size as every other method. Apart from these, all comparison methods were retrained from scratch under the same experimental environment using the hyperparameters provided in Section 5.1, so as to ensure the comparability and fairness of the experimental results. In this section, the performance of the models on the dataset is analyzed in detail. Through systematic quantitative comparisons, the performance of the models is evaluated on various key metrics in the form of “mean ± standard deviation” across the 38 classes of targets. The mean reflects the overall performance of the model, while the standard deviation reflects the consistency of the model’s performance across different target classes: a smaller standard deviation indicates that the model is less sensitive to differences among target classes and possesses stronger generalization ability. The standard deviations reported in Table 1 should be read only in this sense: they describe the distribution of scores across the 38 target classes and are not intended as a measure of run-to-run reproducibility, which would require repeated training with different random seeds. In addition, to further verify the effectiveness of the proposed model on the ISAR image captioning task, qualitative analysis is also conducted. These evaluation results support the effectiveness of the proposed method.

Table 1 details the performance of all comparison methods on the ISARCap 1.0 test set. From the overall results, ISAR-Mamba attained the highest value on each of the eight metrics in this experiment, with the composite metric S_m_ reaching 62.33%, which is 0.28 score points higher than that of HCNet, the second-best method. This advantage suggests that ISAR-Mamba has better comprehensive capability in balancing n-gram matching precision, semantic relevance, and information completeness.

From the perspective of n-gram matching, the BLEU-1 to BLEU-4 scores of ISAR-Mamba reach 78.17%, 64.66%, 52.26%, and 41.79%, respectively, all surpassing those of comparison methods. Particularly for BLEU-4, ISAR-Mamba exceeds HCNet by 0.12 score points and the classic Adaptive-Attention method by 15.57 score points. BLEU-4 imposes stricter constraints on phrase order and consecutive matching, and this improvement indicates that the captions generated by ISAR-Mamba exhibit higher consistency with the annotated data in terms of local syntactic structure and word selection. Notably, compared with the recently proposed RSIC-GMamba method, ISAR-Mamba attains a gain of 0.91 score points on BLEU-4, suggesting the effectiveness of its specific design on ISAR images.

In terms of semantic relevance and information content, the METEOR and ROUGE_L scores of ISAR-Mamba reach 38.19% and 57.02%, respectively, higher than those of the second-best method HCNet. For the CIDEr metric, ISAR-Mamba achieves 112.32%, which is 0.88 score points higher than HCNet, and 4.16 and 10.83 score points higher than DPPF and CDRE, respectively, which employ the CLIP pretrained model, respectively. This phenomenon may stem from the fact that CLIP is trained on natural images, and its visual representations primarily encode natural semantics such as color, texture, and object contours, resulting in a distribution shift when applied to ISAR images. CIDEr emphasizes the unique and critical semantic content of an image through TF-IDF weighting. This advantage indicates that ISAR-Mamba is more accurate in capturing the core structural attributes of space targets and is capable of generating more information-rich captions.

From the perspective of performance stability, the standard deviation of ISAR-Mamba on CIDEr is only 2.18%, which is lower than that of HCNet, CSSA, and DPPF, indicating that its performance has a relatively consistent distribution across the 38 target classes and can maintain stable caption quality for targets with markedly different structural complexity. For the comprehensive metric S_m_, ISAR-Mamba achieves a standard deviation of 5.39%, which is comparable to most methods, maintaining a high level of performance stability. Considering that the asymmetric patch partitioning enables the two streams to cover different physical dimensions, the model can reduce performance fluctuations caused by the absence of features from a single stream across different target categories through complementary information.

**Figure 6:**
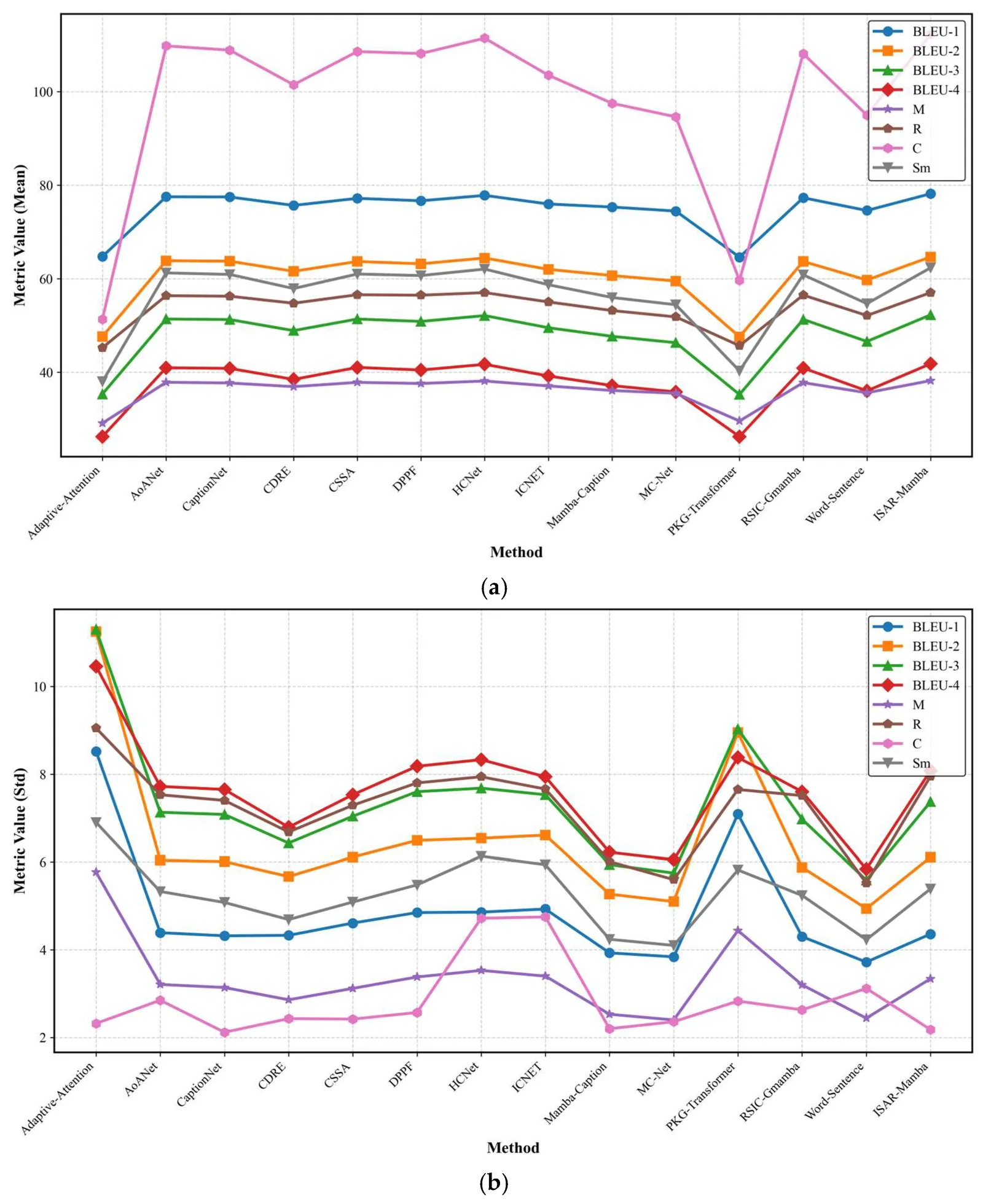
Performance comparison line charts. (**a**) Comparison of the mean values of different methods; (**b**) comparison of the standard deviations of different methods.

Figure 6 further compares the performance distributions of all methods using line charts. Figure 6a shows that the mean line of ISAR-Mamba is higher than those of other methods on most metrics. Figure 6b compares the standard deviations, indicating that ISAR-Mamba also maintains favorable performance, particularly on CIDEr, where its value is smaller than that of most comparison methods. These visualization results corroborate the conclusions of the quantitative statistics and demonstrate the dual advantages of ISAR-Mamba in both accuracy and robustness.

**Figure 7:**
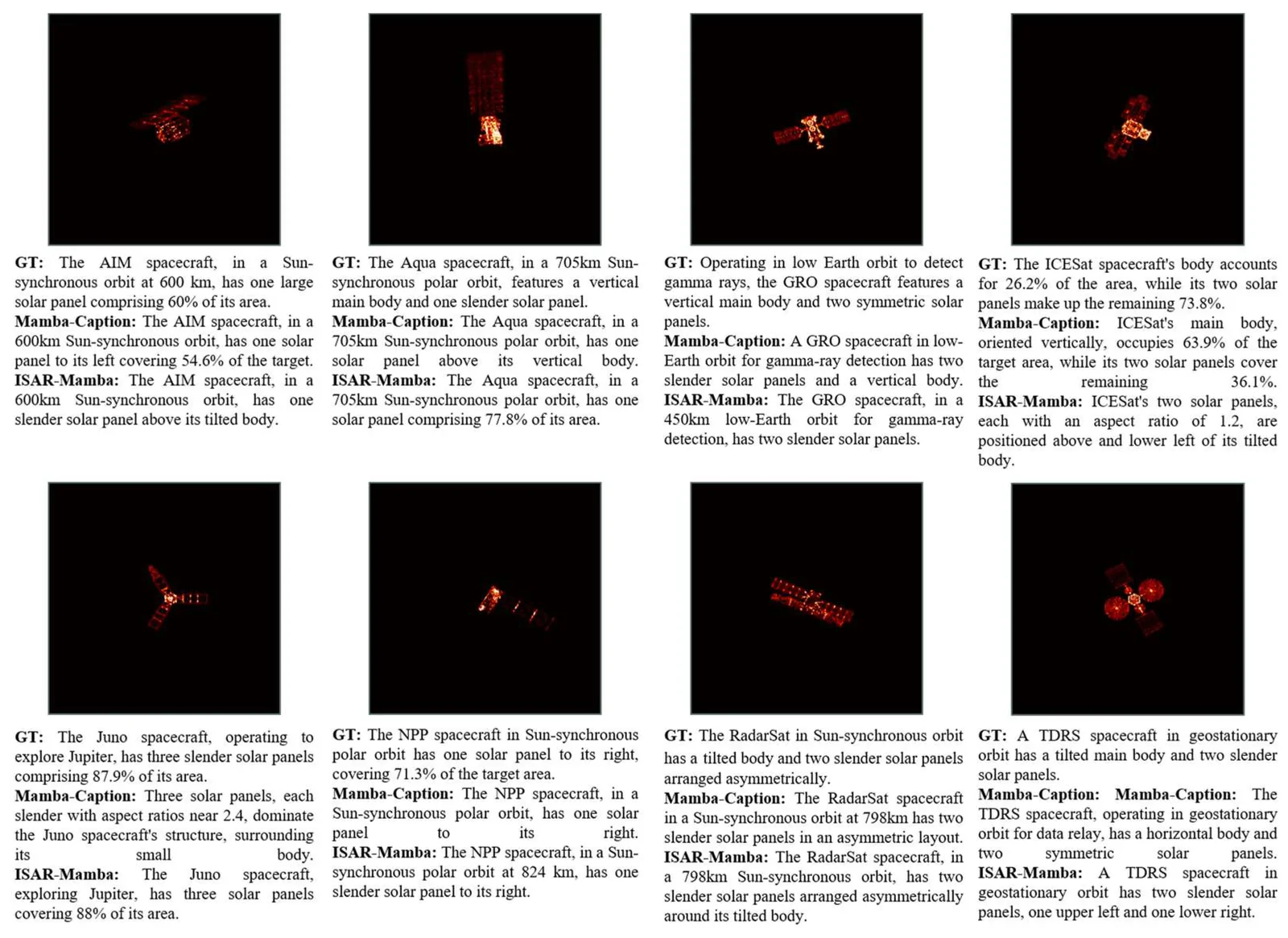
Examples of captions generated by the Mamba-Caption and the proposed ISAR-Mamba, as well as the corresponding ground-truth (GT) captions.

To intuitively evaluate the generation quality, typical samples from the test set were also selected for qualitative analysis. As shown in Figure 7, the captions generated by ISAR-Mamba are consistent with the ground-truth annotations in terms of target type, component composition, and spatial relationships, accurately describing the number of solar panels, the orientation of the main body, and the relative positions between the solar panels and the main body. For targets with complex structures and dense scattering points, the model still maintains coherent and logical captions without omitting key components or inverting spatial relationships. In contrast, comparison methods are more prone to errors such as misjudging the number of solar panels and providing vague descriptions of the main body orientation. The qualitative analysis results corroborate the quantitative metrics, further verifying the effectiveness and robustness of ISAR-Mamba in the ISAR image captioning task, while also suggesting the usability of the ISARCap 1.0 dataset.

### 5.4. Efficiency Comparison

In addition to model accuracy, model efficiency is also a key dimension for evaluating model performance. Parameter count (Params), floating point operations (FLOPs) and inference time are the core metrics in this regard. Specifically, Params reflect the spatial complexity and training requirements of the model, while FLOPs reflect the temporal complexity and computational cost; together, they form a quantitative evaluation system for model efficiency. Inference time refers to the average inference time per sample, i.e., the mean of the inference times over all samples in the test set. To ensure experimental consistency, the model efficiency evaluation was conducted under the same hardware environment as the accuracy experiments in Section 5.3.

**Figure 8:**
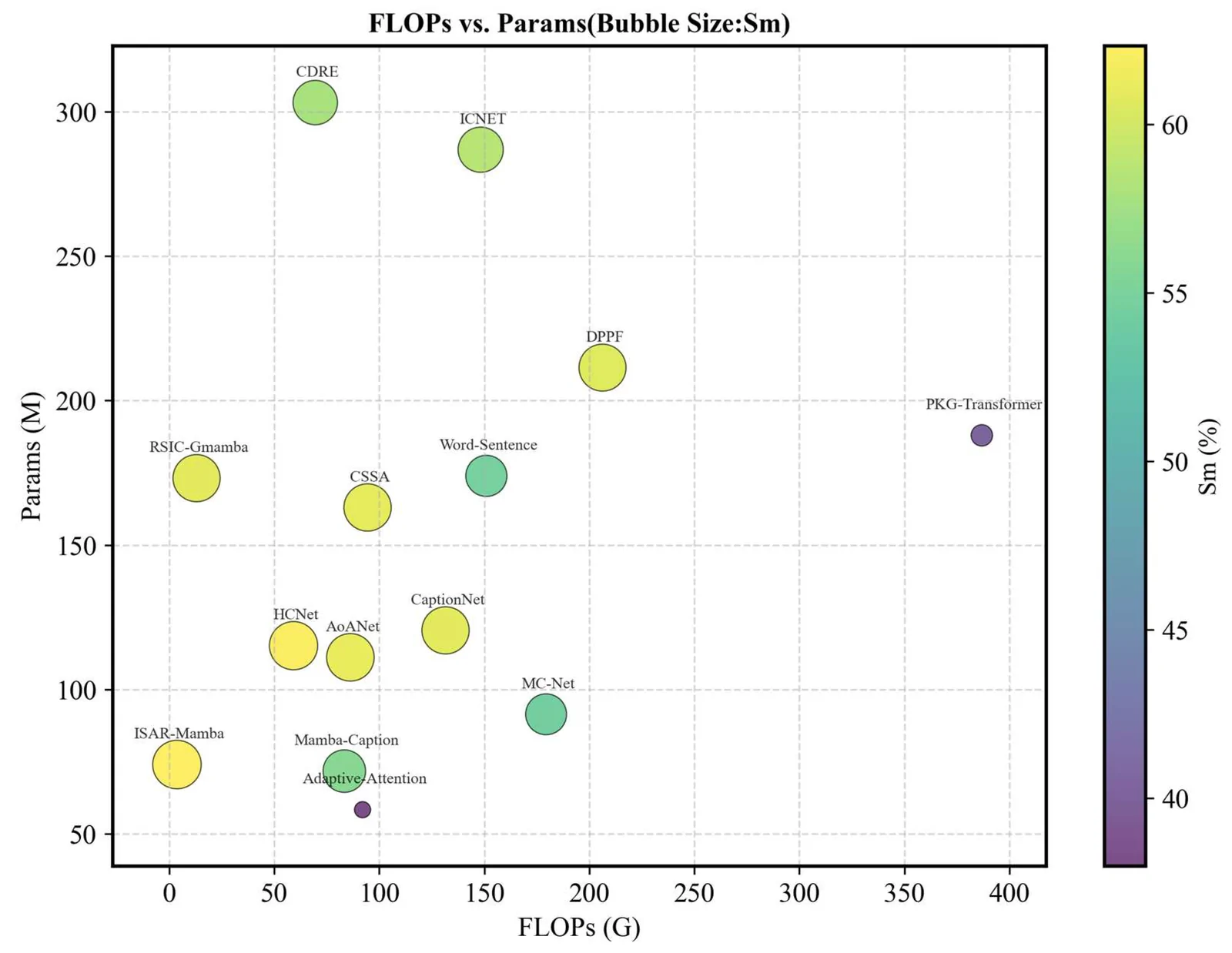
FLOPs–Params scatter plot. The horizontal axis represents FLOPs (G), and the vertical axis represents Params (M). The bubble size is proportional to the S_m_ (%).

Table 2 systematically presents the quantitative comparison results of model efficiency across different methods, while Figure 8 intuitively illustrates the trade-off between accuracy and efficiency through a FLOPs–Params scatter plot. Since the S_m_ metric can comprehensively evaluate model accuracy, only the S_m_ metric is adopted for simplicity.

As can be observed from Table 2 and Figure 8, the relationship between model accuracy and efficiency is not a simple positive correlation, and different technical routes exhibit significant differences in computational cost, storage overhead, and inference latency. ISAR-Mamba achieves the highest S_m_ metric (62.33%) and the lowest computational complexity among the compared methods. This result indicates the structural advantage of SSMs in linear-complexity modeling.

First, in terms of parameter count, ISAR-Mamba has 73.97 M parameters, which is smaller than the majority of Transformer-based comparison methods. For instance, the parameter counts of ICNET and CDRE reach as high as 286.82 M and 303.14 M, respectively, which are approximately 3.9 and 4.1 times that of ISAR-Mamba. The parameter counts of PKG-Transformer and DPPF also reach 187.93 M and 211.38 M, respectively. Some methods with smaller parameter counts, such as Adaptive-Attention (58.34 M), have a smaller model size, but their S_m_ metric is only 37.97%. ISAR-Mamba achieves the highest captioning accuracy with a relatively compact model scale, benefiting from the structural efficiency of its dual-stream asymmetric encoder and hierarchical spatial summarization mechanism—by concentrating limited parameters on modeling effective scattering regions rather than redundant global self-attention computation, it not only avoids the common parameter redundancy in Transformers but also reduces the risk of overfitting. Compared with the Mamba-based methods Mamba-Caption (71.70 M) and RSIC-GMamba (173.11 M), the parameter count of ISAR-Mamba is higher than that of Mamba-Caption but nearly 100 M lower than that of RSIC-GMamba, indicating that the additional parameters introduced by the dual-stream architecture and gating mechanism are reasonable and controllable.

Second, in terms of computational cost, the number of FLOPs for ISAR-Mamba is 3.72 G, the lowest among all compared methods. This value is approximately 71.5% lower than that of RSIC-GMamba (13.06 G), which has the second-lowest FLOPs, and is far lower than those of methods with other architectures—for example, HCNet at 59.22 G, CSSA at 94.46 G, AoANet at 86.28 G, and PKG-Transformer and DPPF are as high as 386.84 G and 206.30 G, respectively. HCNet, as the comparison model with accuracy closest to ISAR-Mamba, has FLOPs approximately 15.9 times that of ISAR-Mamba. DPPF, which employs the CLIP pretrained model, also has a computational cost as high as 55.5 times that of ISAR-Mamba. The reason ISAR-Mamba achieves this advantage is that its core operations are based on selective SSMs, where the state update complexity along each scanning path is linear with respect to the sequence length. Although the dual-stream scanning along the range and azimuth dimensions increases the number of sequences, the number of tokens in each sequence is far smaller than the quadratic complexity of a full-image flattening. In addition, the gating operation in SAGM and the summary token insertion in HSS are both lightweight operations that do not introduce significant additional multiplication computations. Therefore, ISAR-Mamba maintains high accuracy while reducing computational overhead by more than an order of magnitude compared with most Transformer-based baseline methods.

Finally, in terms of inference time, the average inference time of ISAR-Mamba is 9.36 ms, which is at a relatively high level among all methods. In comparison, the inference time of HCNet is only 3.38 ms, AoANet is 3.92 ms, RSIC-GMamba is 4.51 ms, and DPPF is 4.72 ms. This phenomenon may be attributed to multiple factors. Specifically, ISAR-Mamba contains two independent encoding streams, each of which employs bidirectional scanning, and each encoding block incorporates the gating operation of SAGM and the summary token interaction of HSS. These operations introduce a large number of sequence-level kernel calls and synchronization overhead. In the current deep learning framework implementation, small-scale matrix operations and token-wise gating cannot exploit the parallel computing capability of GPUs in the same way as large matrix multiplications, resulting in an actual runtime that does not decrease proportionally with the reduction in FLOPs. Specifically, the following timing protocol was applied identically to every compared method: all methods were evaluated on the same server; the batch size on the test set was 512; 5 warm-up batches were executed before measurement; GPU synchronization was performed before and after each timed batch; the measurement was repeated 10 times over the full test set. The reported inference time is the mean per-sample latency, i.e., the total measured time divided by the number of test samples. The key values of ISAR-Mamba are as follows: the 50th-percentile (P50) batch latency was 4.48 s, the 90th-percentile (P90) batch latency was 4.53 s, the 99th-percentile (P99) batch latency was 12.40 s, the throughput was 106.61 samples/second, and the peak memory usage was 13.58 GB.

ISAR-Mamba achieves the highest captioning accuracy with the lowest computational cost. Its parameter count is also relatively low. Overall, the model shows a favorable accuracy-efficiency trade-off. Although the inference time is longer, this is a necessary overhead introduced by the dual-stream asymmetric architecture and the hierarchical visual prefix. Among the compared methods, ISAR-Mamba obtained the highest reported captioning scores in this experiment with the lowest computational complexity. These results suggest that ISAR-Mamba is computationally efficient in this comparison.

### 5.5. Ablation Experiments

To systematically verify the contributions and synergistic effects of each core module of ISAR-Mamba to the overall performance, a series of ablation experiments are designed in this section. The main advantages of ISAR-Mamba lie in DSAE, SAGM, and HSS. DSAE obtains complementary visual features through asymmetric dual-stream encoding, SAGM regulates patch information propagation through learnable gating, and HSS perceives spatial features at different depths and positions to guide text generation. Therefore, this section focuses on these three components in the ablation experiments. All ablation experiments are conducted under the same hardware environment and hyperparameter configuration, except for the ablated components, so as to ensure fairness. A model without these three designs is used as the baseline for comparison, and Table 3 presents the experimental results of ablating each module.

From the results of the single-module variants, all three modules contribute positively to the performance of the baseline model, but the magnitude of improvement differs among them. The baseline model achieves an S_m_ of 54.30%. When only DSAE is introduced, S_m_ increases to 57.18%, a gain of 2.88 score points over the baseline; when only SAGM is introduced, S_m_ reaches 57.26%, a gain of 2.96 score points; and when only HSS is introduced, S_m_ is 56.35%, a gain of 2.05 score points. These results indicate that SAGM provides the highest gain as a single module among the three, DSAE provides a similar gain, while the independent gain of HSS is relatively low. The core role of HSS is to distill the visual token sequence into a structured prefix, which depends more heavily on the quality of the encoder output features. Therefore, when SAGM or DSAE is absent, the effective information that the summary tokens can integrate may be somewhat limited, which can partly explain the relatively low independent gain of HSS.

From the results of the two-module combinations, the S_m_ of all three pairwise combinations is higher than that of any single-module variant, indicating a positive complementary relationship among the modules. Specifically, the “DSAE + SAGM” combination reaches 60.20%, the “SAGM + HSS” combination reaches 59.31%, and the “DSAE + HSS” combination reaches 59.24%. “DSAE + SAGM” is the highest-scoring two-module combination in this experiment, further improving by 2.94 score points over the best single-module variant. This indicates that the complementary physical-dimension features extracted by the asymmetric dual-stream encoding provide richer decision cues for the gating mechanism, enabling it to identify effective scattering regions more accurately and thereby achieve a higher performance gain.

Although the “SAGM + HSS” combination achieves a slightly lower S_m_ than “DSAE + SAGM”, its parameter count is only 52.47 M and its FLOPs are 1.86 G, compared with 72.4 M parameters and 3.71 G FLOPs for “DSAE + SAGM”. In other words, without dual-stream encoding, the combination of sparse gating and hierarchical summarization can already capture most of the performance gains at a lower computational cost. This phenomenon suggests that the value of DSAE is more evident when it works together with other modules: when used independently, it brings only a 2.88 score point gain, but it provides higher-quality dual-dimensional visual foundations for SAGM and HSS, thereby indirectly amplifying the effects of the latter two. Comparing the two-module combinations with the full model, the full model further improves by 2.13 and 3.02 score points over “DSAE + SAGM” and “SAGM + HSS”, respectively. This indicates that using all three modules simultaneously brings additional performance gains, and the contributions of the modules are not entirely redundant.

From the perspective of computational overhead, the introduction of DSAE increases the parameter count and FLOPs, because the dual-stream structure requires two independent sets of patch embedding and encoding branches. At the same time, the corresponding accuracy improvement is also notable. SAGM, with only a small increase in parameters, effectively suppresses the interference of empty patches through the gating mechanism, achieving a relatively large accuracy improvement at a low additional computational cost. HSS, although adding a certain number of parameters, introduces almost no additional FLOPs while bringing a gain of more than 2 score points, indicating a high efficiency gain in this setting. The inference time of the complete model is higher than those of the other variants, which is a reasonable overhead jointly introduced by the dual-stream encoding and the summary prefix.

**Figure 9:**
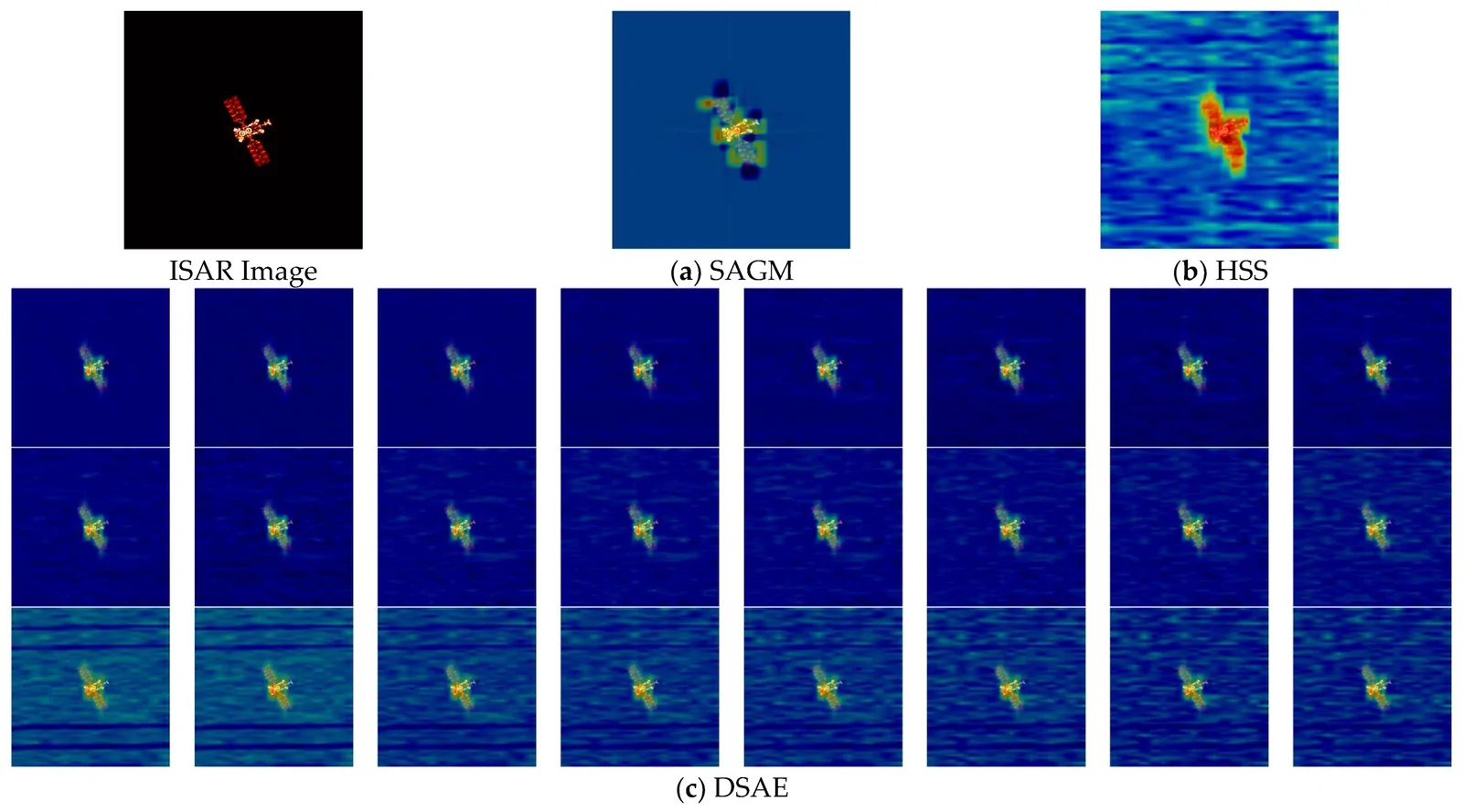
Visualization heatmaps, with intensity increasing from cool to warm colors. (**a**) Distribution of gating scores for each patch; (**b**) similarity distribution between summary tokens and image tokens; (**c**) visualization results of features at each layer of the dual-stream encoder, shown in sequence.

To more intuitively verify the actual functions of each module, visualization analyses are conducted for the gating behavior of SAGM, the dual-stream features of DSAE, and the summary features of HSS, as shown in Figure 9. Figure 9a presents the gating score heatmaps of different patches in SAGM. It can be observed that, after training, the gating scores exhibit a spatial distribution highly concentrated on the strong scattering regions of the target: the gating values are higher on patches corresponding to the main body and solar panels, while they are lower on background and weak scattering regions. This phenomenon indicates that SAGM can automatically distinguish informative and redundant patches based on scattering energy priors and learnable features, thereby concentrating the limited state capacity of the SSM on effective visual regions. In the ablation experiments, the performance degradation after removing SAGM further confirms the critical role of the gating mechanism in suppressing the interference from empty patch information.

Figure 9b presents the similarity heatmaps between summary tokens and image tokens for HSS. It can be observed that each summary token exhibits higher similarity with the image tokens within its corresponding spatial region, indicating that summary tokens can effectively aggregate the local visual information of their own group. Meanwhile, summary tokens also show certain similarity with some strong scattering points in distant regions, suggesting that they also perceive global context through bidirectional SSM scanning. In the ablation experiments, the removal of HSS reduces S_m_ by 2.13 score points, further verifying the important contribution of hierarchical summary tokens to improving the visual-semantic coherence of text generation.

Figure 9c presents the fused feature heatmaps of the range stream and the azimuth stream at different encoder layers for DSAE. These heatmaps are obtained by separately computing the output features of the two streams and then taking their average, reflecting the joint spatial distribution of the dual-stream features. It can be seen that, in the shallow encoder layers, the high-response regions are scattered around the target body and major components. As the network deepens, the responses gradually concentrate toward the target center while preserving the details of edges and strong scattering points. This trend indicates that, through complementary asymmetric dual-stream encoding, DSAE can progressively form a visual representation from local to global and from detail to structure at different depths. The ablation experiments have demonstrated that removing DSAE degrades model performance, confirming the necessity of the dual-stream design. As the layers deepen, the model extracts some invalid features in the background of the image, which appear as noise in the heatmaps, indicating that even though SAGM suppresses the information flow of invalid patches, the information from invalid patches still participates in state updates after multi-layer SSM processing, thereby affecting feature quality.

In summary, DSAE, SAGM, and HSS respectively improve the visual encoding and cross-modal interaction quality of ISAR image captioning from three aspects: asymmetric encoding of physical dimensions, sparsity-aware feature filtering, and hierarchical visual memory. The three modules promote and synergize with each other, jointly constituting the core performance source of ISAR-Mamba.

### 5.6. Further Analysis and Discussion

To validate the usability of the ISARCap 1.0 dataset in supporting a broader range of downstream tasks beyond image captioning, and to further examine the visual representation capability of ISAR-Mamba, this section employs the generated caption texts as a source of structured information and conducts an exploratory investigation of two caption-derived analysis tasks: caption-based target category extraction and solar panel counting. The former assesses the model’s discriminative ability at the target-category semantic level, while the latter focuses on the model’s perceptual accuracy regarding component-level attributes. Through these two tasks, evaluation can be performed at different granularities on whether the annotation information of ISARCap 1.0 can be effectively transferred to other visual understanding scenarios.

#### 5.6.1. Caption-Based Target Category Extraction

Space target recognition is one of the most fundamental tasks in ISAR image interpretation, aiming to determine the class of a target based on its electromagnetic scattering characteristics. Although existing recognition methods have already achieved high accuracy, whether an image captioning model can correctly express the target category in its generated captions remains a question worth exploring. In the ISARCap 1.0 dataset presented in this paper, target type is a core semantic element of the target-level captions. Therefore, class information is extracted from the caption texts generated by each comparison method in Section 5.3, taken as the extracted category of the model, and compared with the ground-truth class labels. The performance of different captioning models on the caption-based category extraction task is thereby evaluated.

Specifically, a rule-based and keyword-matching class extractor is designed. For each test sample, the class name among the 38 space target classes in the dataset is searched for in the caption generated by the model. If the caption contains a certain class name, that class is taken as the predicted class for the sample; if the caption contains no class name, or contains multiple inconsistent class names, it is counted as an incorrect extraction. Captions that contain no class name are counted as a false negative for the ground-truth class only. On this basis, the accuracy (Accuracy), macro-average precision (Precision), macro-average recall (Recall), and macro-average F1 score are calculated as follows:

(18) $$\mathrm{Accuracy}=\frac{N_{\mathrm{correct}}}{N_{total}}$$

(19) $$P_{k}=\frac{TP_{k}}{TP_{k}+FP_{k}}$$

(20) $$R_{k}=\frac{TP_{k}}{TP_{k}+FN_{k}}$$

(21) $$\mathrm{Precision}=\frac{1}{C}\sum_{k=1}^{C}P_{k}$$

(22) $$\mathrm{Recall}=\frac{1}{C}\sum_{k=1}^{C}R_{k}$$

(23) $$F1=\frac{1}{C}\sum_{k=1}^{C}\frac{2\cdot P_{k}\cdot R_{k}}{P_{k}+R_{k}}$$

where $N_{\mathrm{correct}}$ denotes the number of correctly extracted samples, $N_{total}$ denotes the total number of test samples, C denotes the number of classes, and TP, FP, and FN denote true positives, false positives, and false negatives, respectively.

As can be seen from Table 4, ISAR-Mamba achieves a precision of 100%, tied for the highest with CSSA and HCNet; its recall reaches 99.92%, the same as HCNet; its accuracy reaches 99.90%, the same as HCNet and only lower than CSSA’s 99.92% by 0.02 score points; and its F1 score reaches 99.96%, tied with CSSA and HCNet. Overall, ISAR-Mamba, HCNet, and CSSA together constitute the top tier of performance, and the gap with the second tier (such as AoANet and ICNET) is minimal, reflecting that current high-performance captioning models have approached saturation in the expression of class information. Adaptive-Attention and PKG-Transformer perform far worse than the other methods, with accuracies of only 47.54% and 56.72%, respectively. The reason is that these two types of models generate captions of relatively low quality, frequently omitting or misjudging the target class, or using vague references instead of specific class names, which prevents the class extractor from obtaining effective information. This result indicates that caption generation capability directly constrains the accuracy of caption-based category extraction, whereas ISAR-Mamba, through its dual-stream asymmetric encoding and hierarchical spatial summarization, can maintain highly accurate class discrimination during caption generation.

**Figure 10:**
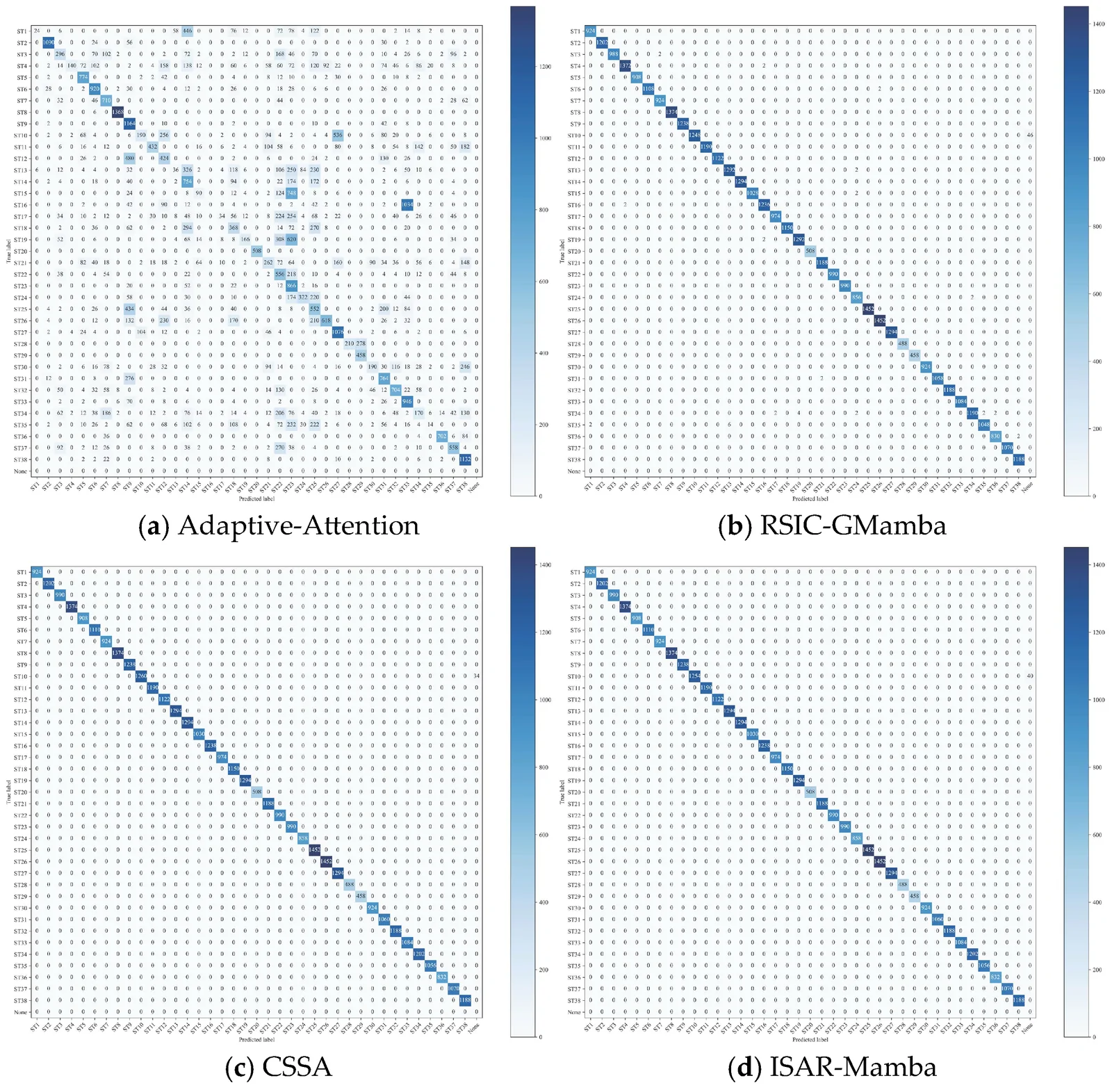
Confusion matrices of some methods.

To further analyze the sources of extraction errors, the confusion matrices of representative methods are presented in Figure 10. Overall, most categories can be correctly extracted, with confusion mainly concentrated among a few structurally similar targets. For example, certain spacecraft with similar appearances and comparable component layouts are occasionally confused with each other in the captions. However, the confusion matrix of ISAR-Mamba is sparser than those of most comparison methods, with fewer errors on easily confused classes, indicating that, through dual-stream asymmetric encoding and hierarchical spatial summarization, it can capture more subtle structural differences and thus maintain stronger class discriminability during the caption generation process.

#### 5.6.2. Solar Panel Counting

Solar panels are one of the most prominent components of space targets, and their number, size, and layout serve as important bases for determining the target type and operational status. In the component-level captions of ISARCap 1.0, the number of solar panels is explicitly annotated, which provides a basis for extracting panel count information from caption texts. The solar panel counting task aims to verify whether a captioning model can accurately perceive and express the number of solar panels in a target, thereby further examining the model’s ability to understand component-level semantic information.

Similarly, solar panel count information is extracted from the captions generated by each model in Section 5.3. Specifically, regular expressions are used to match words or numbers indicating the number of solar panels in the captions; if the count is not explicitly mentioned, it is considered a prediction failure. The predicted panel counts are compared with the ground-truth annotations, and the counting accuracy is calculated. It is defined as the proportion of samples where the predicted number of solar panels exactly matches the ground truth. Table 5 presents the solar panel counting results of all comparison methods.

As can be observed from Table 5, different methods exhibit clear performance stratification on the solar panel counting task. DPPF achieves the highest counting accuracy of 97.97%, closely followed by HCNet and ISAR-Mamba, reaching 97.68% and 97.51%, respectively. These three methods are in the top tier. RSIC-GMamba and CaptionNet also demonstrate relatively good component perception capabilities. In contrast, Adaptive-Attention and PKG-Transformer yield considerably lower counting accuracy, which is consistent with their weak performance on captioning metrics such as BLEU and CIDEr, indicating that these models have insufficient capability in modeling component-level semantic information in ISAR images. The counting accuracy of most of the remaining methods is in the 94–96% range, collectively reflecting a moderate level of performance in expressing component counts.

Although DPPF achieves the highest counting accuracy, its composite captioning metric S_m_ in Section 5.3 is only 60.66%, which is considerably lower than the 62.33% of ISAR-Mamba and the 62.05% of HCNet. This phenomenon indicates that counting accuracy is not entirely consistent with overall caption quality: DPPF obtains strong visual semantic priors through CLIP pretraining, giving it an advantage in explicit component count extraction. However, this advantage is not fully translated into the ability to generate more natural and diverse language captions. Conversely, ISAR-Mamba, without utilizing any pretrained weights, achieves comparable counting performance with low computational complexity while maintaining the highest reported caption quality in this experiment. This suggests that its visual encoding architecture possesses greater comprehensive advantages in supporting multi-task semantic understanding.

Further analysis of counting error samples reveals that most errors occur for targets with complex structures, where the scattering intensity between the solar panels and the main body differs significantly. For example, when the solar panel area is small, some models may omit the panel information in the captions or incorrectly merge the panels with the main body. ISAR-Mamba effectively suppresses the influence of weak scattering regions through the sparsity-aware gating mechanism, while retaining component-level detail features through hierarchical spatial summarization, thereby enabling more accurate identification and counting of solar panels.

In summary, the experimental results of the two caption-derived tasks, caption-based target category extraction and solar panel counting, further demonstrate the potential of the ISARCap 1.0 dataset in supporting multi-task space target interpretation. Image captioning models are capable not only of generating coherent natural language captions, but also of implicitly performing structured semantic understanding tasks such as class discrimination and component attribute perception during the generation process. Through its dedicated design targeting the physical characteristics of ISAR images, ISAR-Mamba achieves leading or comparable performance on both tasks, which further supports the effectiveness of its visual encoding architecture and the information density of the generated captions. The ISARCap 1.0 dataset and the ISAR-Mamba model can provide a reference for future research on multimodal understanding of space targets.

### 5.7. Robustness to Noise

**Figure 11:**
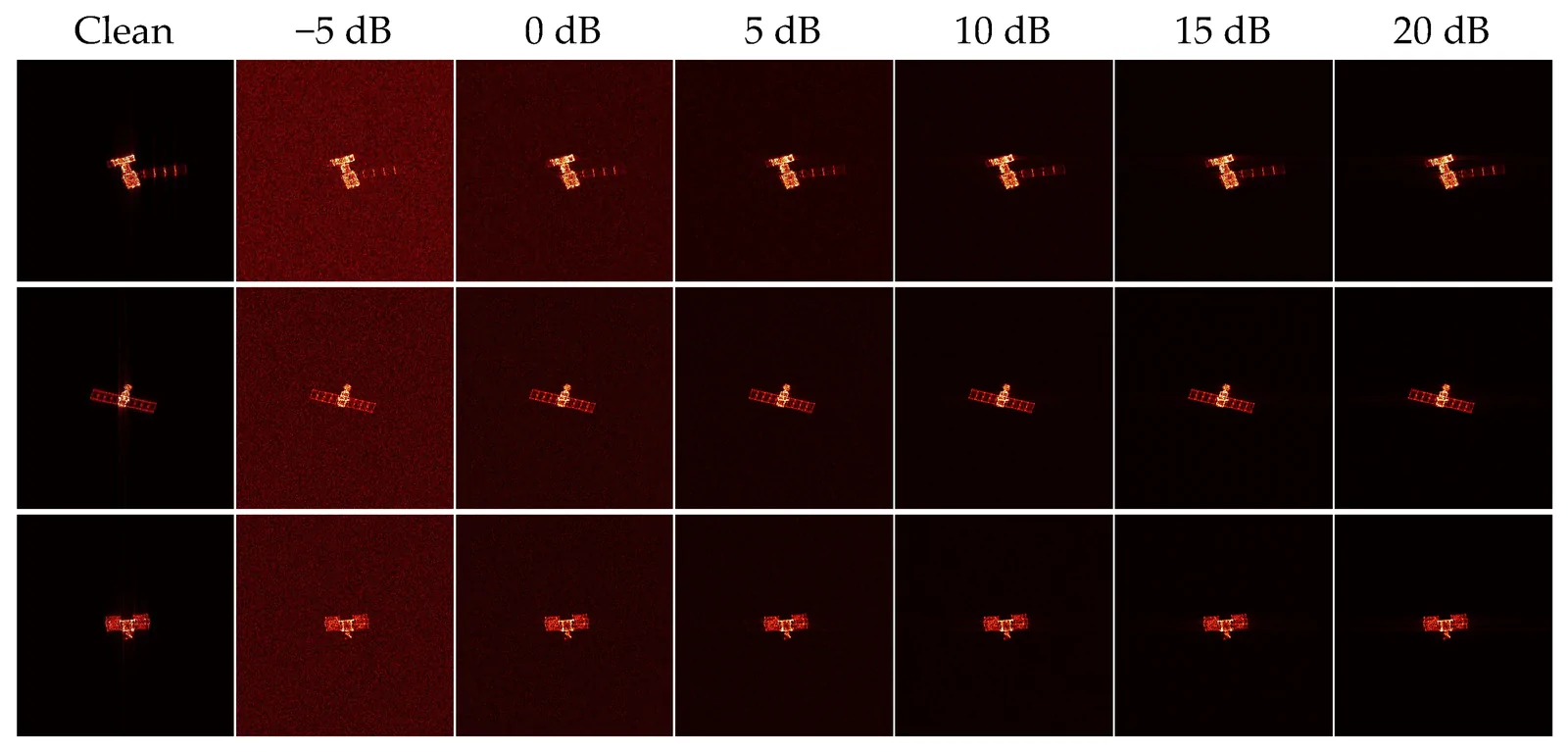
Examples of ISAR images under different SNRs.

In practical scenarios, radar echoes are inevitably affected by noise, causing fluctuations in the intensities of scattering points, thereby affecting the quality of visual features and caption performance. Therefore, to further investigate the model’s performance under non-ideal conditions, this section adds Gaussian white noise of different strengths to the ISAR echoes of the three typical target classes in the dataset, with the Signal-to-Noise Ratio (SNR) set from −5 dB to 20 dB, for a total of six levels. The noisy echoes are then processed through imaging to obtain noisy ISAR images, as shown in Figure 11. This section selects HCNet, which performed best in Section 5.3, as the main comparison method to verify the robustness of ISAR-Mamba to noise. Table 6 shows the performance of HCNet at different SNRs, and Table 7 shows the performance of ISAR-Mamba at different SNRs.

Overall, as the SNR decreases, the performance of both HCNet and ISAR-Mamba generally degrades, indicating that noise negatively affects both visual feature extraction from ISAR images and text generation quality. Comparing the two endpoints, clean and −5 dB, the S_m_ of HCNet drops from 55.50% to 54.33%, a decrease of 1.17 score points, while the S_m_ of ISAR-Mamba drops from 58.67% to 56.47%, a decrease of 2.20 score points. This suggests that under low-SNR conditions, the effective information of scattering points is eroded by noise, and the descriptive model’s ability to perceive target structural details is weakened. The S_m_ achieved by ISAR-Mamba at −5 dB SNR is higher than the S_m_ achieved by HCNet on clean images. In other words, the description quality generated by ISAR-Mamba under the strongest noise interference is better than the level that HCNet can achieve under noise-free conditions. This comparison is limited to the three target classes and six SNR levels covered in this experiment, and its generalizability remains to be validated under broader data conditions.

From the perspective of the BLEU metrics, both models show relatively small degradation in BLEU-1: HCNet drops from 78.33% on clean images to 77.11% at −5 dB, a change of 1.22 score points, while ISAR-Mamba drops from 80.02% to 78.53%, a change of 1.49 score points. Compared with higher-order n-gram metrics, lower-order n-grams are less sensitive to noise, because BLEU-1 mainly measures word-level matching. Even if some scattering details are lost due to noise, the model still tends to generate text containing high-frequency standard vocabulary, thereby maintaining a relatively high BLEU-1 score. In contrast, BLEU-4 imposes stronger constraints on phrase order and consecutive matching, so the loss of detailed information caused by noise has a more pronounced impact on BLEU-4: HCNet drops by 2.08 score points, while ISAR-Mamba drops by 2.85 score points. Although the decrease is larger for ISAR-Mamba, this phenomenon should be understood in the context of its performance under clean conditions. Its BLEU-4 baseline under clean conditions is higher, so the noise weakens its relative advantage over the comparison method rather than pulling its performance below that of the comparison method.

On the CIDEr metric, the two models exhibit different degradation patterns. HCNet’s CIDEr drops from 76.26% on clean images to 74.70% at −5 dB, a decrease of 1.56 score points, with relatively small overall fluctuation. The CIDEr of ISAR-Mamba is more variable: it reaches 85.44% at 10 dB, further rises to 87.04% at 15 dB, but falls back to 83.19% on clean images and drops to 79.76% at −5 dB. This non-monotonic variation may stem from the combined effect of two factors. On the one hand, moderate noise may prompt the model to generate conservative descriptions that are closer to the sentence patterns of the reference annotations, thereby achieving higher matching scores under the weighted mechanism of CIDEr. On the other hand, clean images preserve complete scattering details, and the model tends to generate descriptions containing more variations in specific attributes. The n-gram overlap between these descriptions and the reference annotations may actually be lower than that of templated conservative outputs.

Both models show relatively limited variation in METEOR and ROUGE_L. These two metrics are less sensitive to word order than BLEU-4, and less sensitive to the weighting of rare words than CIDEr, so their responses to changes in the SNR are relatively mild.

A cross-comparison reveals that ISAR-Mamba outperforms HCNet on all metrics at all SNR levels. Under clean conditions, the S_m_ of ISAR-Mamba is 3.17 score points higher than that of HCNet; at −5 dB, this gap narrows to 2.14 score points. This indicates that as noise increases, the performance advantage of ISAR-Mamba over HCNet narrows somewhat, which is consistent with the inference that ISAR-Mamba relies more heavily on detailed scattering information. When scattering points are weakened by noise, the refined feature extraction pathway designed in ISAR-Mamba may be affected more noticeably than in HCNet.

Another noteworthy point is the distribution of the standard deviation of ISAR-Mamba under different SNR conditions. Under clean conditions, the standard deviation of ISAR-Mamba on BLEU-4 is 4.15%, and on CIDEr it is 3.41%; under −5 dB conditions, these values are 8.06% and 5.87%, respectively. This phenomenon indicates that noise not only lowers the overall mean performance but also exacerbates the dispersion of performance across different categories. HCNet shows a certain advantage in this regard. A possible reason for this phenomenon is that the SAGM of ISAR-Mamba can stably identify and suppress empty patches on clean images, whereas under strong noise conditions, noise may be partially misidentified as weak scattering signals, thereby affecting the stability of gating decisions.

In summary, within the range of noise levels covered, ISAR-Mamba maintains an advantage over HCNet across all metrics. However, the model’s behavior under noisy conditions and its relationship with other modules still require further investigation under broader data and degradation conditions. The S_m_ under clean conditions in this section differs from the 38-class results in Table 1, because the noise experiments were conducted only on three typical target classes.

## 6. Conclusions

To address the lack of natural language caption generation capability in ISAR image interpretation, this paper constructs ISARCap 1.0, the first ISAR image captioning dataset for space targets, and proposes the ISAR-Mamba model. ISARCap 1.0 contains 38 classes of space targets, 102,965 images, and 514,825 image–text pairs. Through hierarchical template-based annotation, prompt standardization, and manual correction, it achieves both semantic accuracy and linguistic diversity. ISAR-Mamba incorporates three key technical designs: DSAE adapts to the physical anisotropy between the range and azimuth dimensions of ISAR images; SAGM effectively suppresses the interference of empty patches on state updates and directs limited state capacity toward effective scattering regions; HSS extracts structured visual prefixes from different depths and spatial regions, alleviating the visual information bottleneck of the decoder during generation. Experiments show that ISAR-Mamba obtained the highest reported captioning scores on ISARCap 1.0 among the compared methods and has the lowest computational complexity. The results also support the usability of the dataset and the model on two caption-derived tasks, namely caption-based target category extraction and solar panel counting.

This work still has the following limitations. First, the ISAR images in the dataset are all generated through simulation, leading to a domain gap with real measured ISAR images; the generalization ability of the model on measured data remains to be verified. Second, although the caption texts have been manually corrected, they are overall derived from hierarchical templates and LLM rewriting, and still lag behind natural image captions in terms of expressive freedom and stylistic diversity. Third, the inference time of ISAR-Mamba is higher than that of some baselines, as the dual-stream encoding and summary prefix introduce additional computational overhead. Fourth, the model is trained with the standard cross-entropy loss, without incorporating optimization strategies such as CIDEr optimization or reinforcement learning for improving generation quality. Fifth, more in-depth experiments are still lacking.

Future work will be carried out in the following directions. First, ISARCap 1.0 will be extended to real measured ISAR images, and more target types and imaging conditions will be incorporated to build an evaluation benchmark closer to practical applications. Second, contrastive learning or CIDEr-based optimization methods will be introduced to further improve the naturalness and accuracy of the generated captions. Third, model efficiency will be optimized through knowledge distillation, model pruning, and parallel inference to enhance real-time deployment capability while maintaining accuracy. Fourth, the evaluation will be extended by repeating the training of the proposed model and of the strongest baselines with multiple random seeds, so that run-level confidence intervals and formal paired tests can be reported in place of the present cross-class dispersion statistics; by evaluating the models under unseen attitudes, held-out target classes and different imaging conditions; and by ablating the internal design choices of the three modules. Finally, based on ISARCap 1.0, more downstream tasks such as visual question answering, target detection, and attribute recognition will be explored to promote the further development of multimodal intelligent interpretation technology for space targets.

## Figures and Tables

**Table 1:** Comparison results on ISARCap 1.0. Results are reported as percentage (%).

Method	B-1	B-2	B-3	B-4	M	R	C	S_m_
Adaptive-Attention	64.75 ± 8.52	47.62 ± 11.25	35.32 ± 11.30	26.22 ± 10.46	29.08 ± 5.77	45.23 ± 9.05	51.34 ± 2.32	37.97 ± 6.90
AoANet	77.52 ± 4.39	63.83 ± 6.04	51.36 ± 7.13	40.93 ± 7.72	37.83 ± 3.21	56.36 ± 7.53	109.80 ± 2.85	61.23 ± 5.33
CaptionNet	77.47 ± 4.32	63.73 ± 6.01	51.25 ± 7.08	40.80 ± 7.65	37.68 ± 3.14	56.24 ± 7.40	108.88 ± 2.12	60.90 ± 5.08
Word-Sentence	74.58 ± 3.72	59.70 ± 4.94	46.56 ± 5.59	35.99 ± 5.84	35.58 ± 2.44	52.11 ± 5.52	94.98 ± 3.12	54.66 ± 4.23
MC-Net	74.46 ± 3.84	59.49 ± 5.10	46.30 ± 5.75	35.77 ± 6.05	35.47 ± 2.40	51.82 ± 5.60	94.62 ± 2.36	54.42 ± 4.10
PKG-Transformer	64.60 ± 7.09	47.55 ± 8.95	35.21 ± 9.03	26.22 ± 8.38	29.55 ± 4.44	45.65 ± 7.65	59.66 ± 2.83	40.27 ± 5.82
HCNet	77.81 ± 4.86	64.42 ± 6.54	52.10 ± 7.68	41.67 ± 8.33	38.10 ± 3.53	57.01 ± 7.94	111.44 ± 4.72	62.05 ± 6.13
ICNET	75.97 ± 4.93	61.99 ± 6.61	49.50 ± 7.53	39.18 ± 7.94	37.05 ± 3.40	55.02 ± 7.66	103.53 ± 4.75	58.70 ± 5.94
Mamba-Caption	75.32 ± 3.93	60.66 ± 5.27	47.66 ± 5.94	37.12 ± 6.22	36.07 ± 2.53	53.17 ± 6.01	97.51 ± 2.20	55.97 ± 4.24
RSIC-GMamba	77.30 ± 4.30	63.68 ± 5.88	51.28 ± 6.97	40.88 ± 7.60	37.76 ± 3.20	56.48 ± 7.51	108.12 ± 2.63	60.81 ± 5.24
DPPF	76.66 ± 4.85	63.18 ± 6.49	50.85 ± 7.60	40.45 ± 8.18	37.57 ± 3.38	56.46 ± 7.80	108.16 ± 2.57	60.66 ± 5.48
CSSA	77.18 ± 4.61	63.67 ± 6.11	51.36 ± 7.04	41.01 ± 7.53	37.81 ± 3.12	56.56 ± 7.29	108.58 ± 2.42	60.99 ± 5.09
CDRE	75.67 ± 4.33	61.57 ± 5.67	48.88 ± 6.43	38.44 ± 6.79	36.89 ± 2.86	54.74 ± 6.68	101.49 ± 2.43	57.89 ± 4.69
**ISAR-Mamba**	**78.17 ± 4.36**	**64.66 ± 6.11**	**52.26 ± 7.37**	**41.79 ± 8.08**	**38.19 ± 3.34**	**57.02 ± 7.95**	**112.32 ± 2.18**	**62.33 ± 5.39**

**Table 2:** Comparison of efficiency and accuracy of different models.

Method	Params (M)	FLOPs (G)	Inference Time (ms)	S_m_ (%)
Adaptive-Attention	58.34	92.08	5.03	37.97
AoANet	111.09	86.28	3.92	61.23
CaptionNet	120.42	131.58	5.66	60.90
Word-Sentence	173.90	151.00	7.37	54.66
MC-Net	91.37	179.44	5.03	54.42
PKG-Transformer	187.93	386.84	21.45	40.27
HCNet	115.13	59.22	3.38	62.05
ICNET	286.82	148.30	8.01	58.70
Mamba-Caption	71.70	83.40	5.59	55.97
RSIC-GMamba	173.11	13.06	4.51	60.81
DPPF	211.38	206.30	4.72	60.66
CSSA	162.97	94.46	6.42	60.99
CDRE	303.14	69.56	4.64	57.89
**ISAR-Mamba**	**73.97**	**3.72**	**9.36**	**62.33**

**Table 3:** Ablation Results.

Method	DSAE	SAGM	HSS	Params (M)	FLOPs (G)	Inference Time (ms)	S_m_ (%)
Baseline	×	×	×	50.30	1.65	1.82	54.30 ± 4.20
	√	×	×	72.35	2.08	7.81	57.18 ± 5.17
	×	√	×	52.46	1.86	5.46	57.26 ± 5.23
	×	×	√	52.06	1.65	2.24	56.35 ± 5.41
	×	√	√	52.47	1.86	2.29	59.31 ± 4.90
	√	√	×	72.40	3.71	8.14	60.20 ± 5.30
	√	×	√	73.17	2.08	8.85	59.24 ± 5.56
	√	√	√	73.97	3.72	9.36	62.33 ± 5.39

**Table 4:** Comparison of caption-based category extraction results on ISARCap 1.0 (%).

Method	Precision	Recall	Accuracy	F1
Adaptive-Attention	55.25	49.35	47.54	45.06
AoANet	99.98	99.90	99.88	99.94
CaptionNet	99.99	99.87	99.84	99.93
Word-Sentence	99.59	99.49	99.45	99.54
MC-Net	99.71	99.50	99.46	99.60
PKG-Transformer	56.21	54.90	56.72	52.96
HCNet	100	99.92	99.90	99.96
ICNET	99.97	99.89	99.88	99.93
Mamba-Caption	99.73	99.62	99.59	99.67
RSIC-GMamba	99.90	99.81	99.79	99.86
DPPF	99.92	99.79	99.76	99.85
CSSA	100	99.93	99.92	99.96
CDRE	99.95	99.88	99.86	99.92
**ISAR-Mamba**	**100**	**99.92**	**99.90**	**99.96**

**Table 5:** Comparison of counting results on ISARCap 1.0 (%).

Method	Accuracy
Adaptive-Attention	79.66
AoANet	95.85
CaptionNet	97.15
Word-Sentence	93.91
MC-Net	94.36
PKG-Transformer	83.55
HCNet	97.68
ICNET	96.11
Mamba-Caption	94.60
RSIC-GMamba	97.44
DPPF	97.97
CSSA	95.51
CDRE	95.05
**ISAR-Mamba**	**97.51**

**Table 6:** Experimental results of HCNet at different SNRs (%).

SNR (dB)	B-1	B-2	B-3	B-4	M	R	C	S_m_
−5	77.11 ± 3.62	65.43 ± 3.81	54.27 ± 4.01	43.31 ± 3.89	38.06 ± 4.62	61.23 ± 4.14	74.70 ± 2.39	54.33 ± 3.76
0	77.34 ± 3.54	65.75 ± 4.00	54.45 ± 4.06	44.48 ± 4.06	38.13 ± 4.74	61.99 ± 4.48	74.72 ± 3.05	54.83 ± 4.08
5	77.62 ± 4.18	66.01 ± 4.31	54.67 ± 4.11	44.60 ± 4.26	38.03 ± 4.57	61.24 ± 4.16	75.39 ± 8.07	54.81 ± 5.27
10	77.96 ± 4.56	66.05 ± 4.56	54.55 ± 4.33	44.39 ± 4.08	38.07 ± 4.69	61.99 ± 4.52	74.68 ± 5.02	54.78 ± 4.58
15	77.91 ± 4.31	66.17 ± 4.61	54.82 ± 4.92	44.75 ± 4.79	38.12 ± 4.58	62.03 ± 4.56	75.46 ± 5.16	55.09 ± 4.77
20	77.94 ± 4.49	66.24 ± 4.80	54.93 ± 4.83	44.91 ± 4.76	38.10 ± 4.69	62.00 ± 4.49	75.74 ± 4.79	55.19 ± 4.68
**clean**	**78.33 ± 3.99**	**66.64 ± 4.30**	**55.35 ± 4.29**	**45.39 ± 4.17**	**38.25 ± 4.47**	**62.09 ± 4.38**	**76.26 ± 2.68**	**55.50 ± 3.92**

**Table 7:** Experimental results of ISAR-Mamba at different SNRs (%).

SNR (dB)	B-1	B-2	B-3	B-4	M	R	C	S_m_
−5	78.53 ± 5.09	66.83 ± 6.27	55.48 ± 7.23	45.57 ± 8.06	38.40 ± 5.55	62.13 ± 7.16	79.76 ± 5.87	56.47 ± 6.66
0	78.55 ± 3.11	66.87 ± 3.45	55.40 ± 3.48	45.46 ± 3.39	38.47 ± 4.50	62.30 ± 4.54	77.98 ± 3.54	56.05 ± 3.99
5	78.47 ± 3.26	66.79 ± 3.63	55.50 ± 3.65	45.61 ± 3.50	38.43 ± 4.49	62.04 ± 4.33	83.02 ± 3.04	57.27 ± 3.84
10	78.75 ± 3.96	67.66 ± 4.85	56.76 ± 5.41	47.12 ± 5.83	38.87 ± 5.14	63.04 ± 5.80	85.44 ± 1.46	58.62 ± 4.56
15	79.40 ± 3.95	67.70 ± 4.18	56.39 ± 4.19	46.50 ± 4.04	38.69 ± 4.52	62.55 ± 4.98	87.04 ± 0.82	58.70 ± 3.59
20	79.45 ± 3.81	68.30 ± 4.75	57.43 ± 5.44	47.93 ± 5.97	39.06 ± 5.05	63.43 ± 6.09	84.02 ± 0.56	58.61 ± 4.42
**clean**	**80.02 ± 3.64**	**68.77 ± 4.07**	**57.81 ± 4.19**	**48.42 ± 4.15**	**39.17 ± 4.56**	**63.91 ± 4.52**	**83.19 ± 3.41**	**58.67 ± 4.16**

## Data Availability

The data that support the findings of this study are available from the corresponding author upon reasonable request.
